# Multi-target approach of Egyptian leek extract in ameliorating depressive-like behavior in rats exposed to chronic unpredictable mild stress

**DOI:** 10.3389/fphar.2025.1621762

**Published:** 2025-08-04

**Authors:** Mai M. S. Mahmoud, Amina E. Essawy, Ahmed A. Soffar, Ahmed H. I. Faraag, Mohamed A. Dkhil, Omar A. Ahmed-Farid, Manal F. El-Khadragy, Ahmed E. Abdel Moniem

**Affiliations:** ^1^ Euro-Mediterranean Program in Neuroscience and Biotechnology, Faculty of Science, Alexandria University, Alexandria, Egypt; ^2^ Zoology Department, Faculty of Science, Alexandria University, Alexandria, Egypt; ^3^ Botany and Microbiology Department, Faculty of Science, Helwan University, Cairo, Egypt; ^4^ Department of Zoology and Entomology, Faculty of Science, Helwan University, Helwan University, Cairo, Egypt; ^5^ Applied Science Research Center, Applied Science Private University, Amman, Jordan; ^6^ Physiology Department, Egyptian Drug Authority, Giza, Egypt; ^7^ Department of Biology, College of Science, Princess Nourah Bint Abdulrahman University, Riyadh, Saudi Arabia; ^8^ Al-Ayen Scientific Research Center, Al-Ayen Iraqi University, An Nasiriyah, Iraq

**Keywords:** depression, Egyptian leek, chronic unexpected mild stress, oxidative stress, neuroinflammation, apoptosis, cerebral cortex

## Abstract

**Introduction:**

Depression is a widespread and debilitating disorder with a complex etiology involving oxidative stress, neuroinflammation, and apoptosis. Given the limitations of current treatments, there is an urgent need for alternative therapeutic approaches. This study evaluated the antidepressant effects of Egyptian leek (Allium ampeloprasum var. kurrat) in rats exposed to chronic unpredictable mild stress (CUMS), a well-established model for studying depression, while exploring the underlying molecular mechanisms. Egyptian leek extract is rich in bioactive compounds, including flavonoids, alkaloids, terpenoids, steroids, and saponins, which are known for their neuroprotective and mood-regulating properties.

**Methods:**

Using liquid chromatography-mass spectrometry (LC-MS) and Fourier-transform infrared spectroscopy (FT-IR), the leek extract was analyzed to identify bioactive compounds. In this study, CUMS-exposed rats were treated with the extract, and their behavior was assessed using the sucrose preference test (SPT), open field test (OFT), and social interaction test (SIT). Concurrently, oxidative stress and antioxidant factors, pro-inflammatory cytokines, and apoptotic proteins in the cerebral cortex were evaluated, and molecular docking analyses were conducted to investigate the extract’s mechanisms of action.

**Results:**

The results demonstrated that treatment with Egyptian leek extract significantly alleviated depression-like behaviors, as evidenced by improved performance in the SPT, OFT, and SIT. Moreover, the leek extract improved oxidative stress parameters, reduced levels of pro-inflammatory cytokines, suppressed NF-κB activation, and promoted neuronal survival by modulating apoptosis-related proteins.

**Discussion:**

These results suggest that the leek extract effectively mitigates oxidative stress, inflammation, and apoptosis, thereby contributing to its overall antidepressant effects.

## 1 Introduction

Depression is a pervasive and heterogeneous mental disorder that negatively impacts both psychological wellbeing and health systems (Li et al., 2024). Globally, it is recognized as a significant public health challenge, with World Health Organization (WHO) prevalence estimates indicating over 280 million affected individuals worldwide (Global Health Data Exchange, 2023). Depression is marked by a consistently low mood, loss of pleasure and interest in daily activities, cognitive impairments, and physical changes such as altered appetite and sleep (Kupferberg and Hasler, 2023), It is not only debilitating but also increases the risk of developing several chronic illnesses (Voinov et al., 2013). Additionally, it is a major contributor to mortality, with over 700,000 deaths annually attributed to suicide, a tragic outcome of severe depression (Global Health Data Exchange, 2023). WHO has also identified depression as the second leading cause of global mortality, after cancer (Tang et al., 2025).

Depression pathogenesis involves various signaling pathways and molecular changes (Guo et al., 2024). It is associated with dysregulation of several cellular and molecular mechanisms, including disruptions in the hypothalamic-pituitary-adrenal (HPA) axis, neurotransmitter imbalances, neuroinflammation, oxidative stress, and impaired neurogenesis and synaptic plasticity (Liu et al., 2020; Liu et al. 2024). Chronic stress plays a central role in initiating a cascade of biological responses that exacerbate the onset and progression of depression. One key mechanism is the neuroinflammatory response, which involves increased expression levels of pro-inflammatory cytokines such as interleukin-1β (IL-1β), interleukin-6 (IL-6), and tumor necrosis factor-α (TNF-α). These cytokines disrupt the HPA axis, interfere with synaptic neurotransmission, and reduce the levels of brain-derived neurotrophic factor (BDNF) (Liu et al., 2024). BDNF is a critical neurotrophic factor for synaptic plasticity, neuronal survival, and emotional regulation; its reduction impairs neuroplasticity, diminishes excitatory neurons, and contributes to depressive symptoms (Correia et al., 2023). Furthermore, depression involves reduced activity of the serotonergic system, particularly through alterations in the activity or expression of the 5-Hydroxytryptamine (serotonin) receptor 1A (5-HT_1A_) in brain areas such as the prefrontal cortex, which is responsible for regulation of mood, emotional processing, and memory (Limón-Morales et al., 2023). Given these interconnected mechanisms, potential treatments for depression may include strategies to upregulate BDNF, block inflammatory cytokine signaling, enhance antioxidant defenses, restore neurotransmitter balance and reverse the deficits, thereby alleviating depressive symptoms.

While there are several conventional treatment options for depression, including antidepressants like selective serotonin reuptake inhibitors (SSRIs), tricyclics (TCAs), serotonin-noradrenaline reuptake inhibitors (SNRIs), and monoamine oxidase inhibitors (MAOIs) (Wang et al., 2025b), up to one-third of individuals with depression fail to respond adequately to these therapies. Moreover, these medications often require a period of six to 8 weeks to demonstrate their effectiveness (Wang X. et al., 2024) and are often associated with a range of side effects (Wang et al., 2025b). These factors contribute to non-adherence and early discontinuation of treatment, which play a key role in depression becoming a chronic condition, creating significant challenges in its management (Alemayehu et al., 2022). Despite the availability of various antidepressant options, the current treatments are far from ideal, with many patients experiencing either inadequate response or intolerable side effects (Wang et al., 2025b). Given the growing prevalence of depression, there is an urgent need for the development of more potent, effective, and safer treatments, based on an in-depth understanding of its pathophysiology.

Promising agents such as medicinal plant extracts (Moragrega and Ríos, 2021), marine natural products (Fakhri et al., 2021), and nanoparticles (Zorkina et al., 2020) can be effective and safer alternatives to depression treatment with low or no side effects compared to currently available depression drugs (Lee and Bae, 2017). Recent studies have validated the health benefits and medicinal value of Egyptian leek (*Allium ampeloprasum* var. *kurrat*). Leek is a green, widely cultivated vegetable belonging to the *Allium* genus (family Alliaceae). Egyptian leek contains a wide range of valuable bioactive compounds known for their therapeutic potentials, such as flavonoids, alkaloids, terpenoid, steroids, and saponins (El-Rehem and Ali, 2013), which demonstrate strong neuroprotective, immunomodulatory, antioxidant and anti-inflammatory (El-Rehem and Ali, 2013; Ammar et al., 2020), activities. Given its ability to modulate immune responses, reduce oxidative stress, and nourish and support brain functions, leek may be an effective treatment for depression. Till now, there has been no thorough research on the effect of leek on depression. Our study aims to investigate the therapeutic potential of the Egyptian leek extract against depression in chronic unexpected mild stress (CUMS)-exposed rats and its regulatory mechanism for modulating neurotransmitters and BDNF levels and mitigating oxidative damage and proinflammatory cytokines.

## 2 Materials and methods

### 2.1 Ethical approval

Institutional review board approval and Ethical approval for this study was obtained from Institutional Review Board (IRB), Alexandria University, Alexandria, Egypt (approval No. AU04231128103).

### 2.2 Leek extract preparation

The leaves of Egyptian leek (*Allium ampeloprasum* var. *kurrat*) were thoroughly rinsed with water to remove any residual soil. The leaves were then juiced using an electric blender (Braun, Germany) and then left for about 48 h in a refrigerator for approximately 48 h to allow for sedimentation and extraction equilibration. After this period, the mixture was filtered using a Buchner funnel and diluted with distilled water (1:1) and stored at −20°C for up to 2 months to preserve its integrity.

### 2.3 Chemical screening

#### 2.3.1 Total phenolics content

To assess the total phenolic content, approximately 400 µL of the aqueous extract (1 mg/mL) was added to 1 mL of Folin–Ciocalteu reagent (10%) in a test tube. After incubating for 5–8 min at 25 °C, 800 µL of sodium carbonate (7.5%) solution (Na_2_CO_3_) was added. The total volume was then adjusted to 10 mL with distilled water. After 2 h incubation period, absorbance was measured at 765 nm versus a blank. The total phenolic content in the leek extract was determined using gallic acid calibration curve as the standard and reported as milligrams of gallic acid equivalents (GAE) per gram of extract (mg GAE/g extract) (Abdel Moneim, 2013).

#### 2.3.2 Total flavonoid content

To assess the total flavonoid content, a volume of 250 µL of the aqueous extract was mixed with 1.5 mL of distilled water. Following this, 90 µL of sodium nitrite (NaNO_2_) solution (5%) was added. The reaction was allowed to proceed for exactly 6 min before the addition of 180 µL of aluminum chloride (AlCl_3_) solution (10%). After an additional 5 min, 600 µL of 1 M sodium hydroxide (NaOH) was added to the mixture. The final volume was brought to 3 mL with distilled water, and the mixture was thoroughly mixed. Absorbance was measured at 510 nm against a blank. Quercetin was used for the calibration curve as standard, and the total flavonoid content in the leek extract was calculated and expressed as milligrams of quercetin equivalents (QE) per gram of extract (mg QE/g extract) (Abdel Moneim, 2013).

#### 2.3.3 Fourier-transform infrared spectroscopy (FT-IR)

A small amount of leek extract was combined with potassium bromide (KBr) in a 1:99 weight ratio and thoroughly ground to achieve a uniform mixture before being finely pulverized. This mixture was analyzed using Thermo Scientific Nicolet 6700 FT-IR spectrometer (Waltham, United States). The FT-IR spectrum was recorded over the wavenumber range of 4,000–400 cm^−1^ at 25°C, capturing the maximum absorption peaks, which indicates the functional groups present in the extract (Ahamad et al., 2023).

#### 2.3.4 Liquid chromatography-mass spectrometry (LC-MS)

For the identification of phytochemicals from Egyptian leek, LC–Electrospray Ionization Mass Spectrometry (ESI-MS) was conducted using both positive and negative ion acquisition modes on a XEVO TQD triple quadrupole mass spectrometer (Waters Corporation, Milford, MA, United States). Chromatographic separation was achieved using an ACQUITY UPLC BEH C18 column (2.1 × 50 mm, 1.7 µm particle size). The flow rate was set to 0.2 mL/min. Gradient elution was carried out using two solvents: water containing formic acid (0.1%) (solvent A) and acetonitrile containing formic acid (0.1%) (solvent B) (Ezghayer et al., 2024). This approach allowed for the effective identification of diverse phytochemicals critical to the pharmacological properties of the extract.

### 2.4 Molecular docking of the leek extract

Molecular docking analysis was conducted to investigate the interactions between bioactive components present in Egyptian leek extract and several target proteins implicated in depression. The bioactive compounds selected included Flavonoids: Quercetin, Quercetin-7-O-rutinoside, Kaempferol 3-glucoside, Luteolin 7-O-glucoside, and Apigenin 7-O-glucoside, Phenolic Acids: Gallic acid and Chlorogenic acid, Amino Acids: Phenylalanine, Tryptophan and L-Isoleucine, Cyclic Nucleotides: cAMP (Cyclic adenosine monophosphate) and Sulfur Compounds: Methyl-L-cysteine, Dipropyl disulfide, Diallyl disulfide, and Allicin. The target proteins analyzed were BDNF, 5-HT_1A_, glial fibrillary acidic protein (GFAP), nuclear factor erythroid 2-related factor 2 (Nrf2), and nuclear factor kappa B (NF-κB). Docking simulations were performed using Schrodinger Maestro 14.0 software. Imipramine was included as a reference antidepressant to compare binding affinities and interaction modes, providing insights into the therapeutic mechanisms of these compounds in depression treatment.

#### 2.4.1 Ligand preparation

The chemical structures of all bioactive compounds and imipramine were retrieved from the PubChem database (https://pubchem.ncbi.nlm.nih.gov). Energy minimization of the compounds was performed to obtain the most stable ligand conformations at the lowest energy using the Macromodel minimization tool in Schrodinger Maestro 14.0 to obtain the most stable ligand conformations at the lowest energy (Mahmoud et al., 2021).

#### 2.4.2 Protein preparation

The complete 3D structure of BDNF was predicted using the AlphaFold tool in ChimeraX 1.9 (Tzfadia et al., 2024). The 3D structures for the 5-HT_1A_, GFAP, Nrf2 and NF-κB were downloaded from the Protein Data Bank (PDB) database (https://www.rcsb.org/) (Mahmoud et al., 2021) using the following accession number PDB: 7E2Y, 6A9P, 7O7B and 7CLI, respectively.

#### 2.4.3 In silico molecular modelling

*In silico* docking was employed to elucidate the mode of action of the studied ligands. Prior to docking, the target protein was properly prepared using the Protein Preparation Workflow tool in Schrödinger’s software suite, ensuring that the protein was biologically relevant and devoid of errors that could compromise the docking results. The SiteMap tool was then used to analyze the protein’s surface and identify potential ligand-binding sites. These predicted sites were critical for generating a receptor grid, which was subsequently used to guide the docking simulations (Mahmoud et al., 2021). The ligands were docked using Glide’s Extra Precision (XP) mode, noted for its high accuracy. Finally, the docking results were visualized and analyzed using Maestro (Osman et al., 2024).

### 2.5 Animals and housing conditions

A total of 30 male Wistar rats weighing 180–200 g was used as experimental animals. The animals were housed in specific pathogen-free conditions at a controlled temperature of 23°C ± 5 °C, maintained under a 12 h/12 h light-dark cycle. They had unrestricted access to a commercial pellet diet and water throughout the 7-day acclimation period before the initiation of the experiment.

### 2.6 Animal grouping and experimental design

**FIGURE 1:**
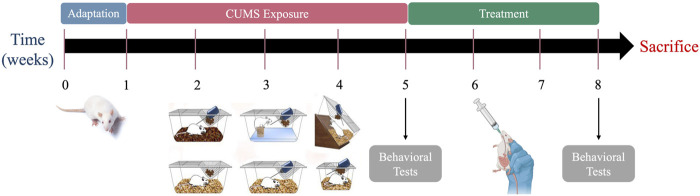
The experimental procedure schedule.

After 1 week of adaptation, rats were randomly divided into five groups, with six rats per group. The experimental groups are Control, Leek, CUMS, CUMS + Leek, and CUMS + Imipramine (Figure 1). Rats in the CUMS groups were housed individually to facilitate the induction of depressive-like symptoms using CUMS model according to previously described protocols (Abelaira et al., 2013; Tian et al., 2015). Over a 4-week period, the CUMS protocol involved daily exposure (in random order) to various stressors, which included: noise exposure for 3 h, water deprivation for 24 h, food deprivation for 24 h, immersion in cold water, social isolation, and reversal of the light/dark cycle. The treatment duration was 3 weeks, with oral administration once per day every morning. In the Leek and CUMS + Leek groups, leek extract [100 mg/kg, orally (p.o.)] was administered (Al-Otaibi et al., 2020). In the CUMS + Imipramine group, imipramine (10 mg/kg, p. o.) was administered (Berk et al., 2020). For the Control and CUMS groups, saline was administered during the same period.

### 2.7 Body weight measurement

The body weight of each rat was recorded once a week, from day 0 until the end of the experiment.

### 2.8 Behavioral assessments

#### 2.8.1 Sucrose preference test (SPT)

Anhedonia, a key symptom of depression (Yin et al., 2021), was assessed through the SPT, in which each rat was provided with two bottles, one containing a sucrose solution (1%) and the other containing tap water, for 4 h. The consumption of sucrose and water was recorded (Jianguo et al., 2019).

#### 2.8.2 Open field test (OFT)

The OFT was performed according to the previously described protocol (Jiang et al., 2023) with some modifications. Each rat was individually introduced to an open-field apparatus (60 × 60 × 40 cm). A camera mounted above the arena was used to track and record the rats’ movements. The arena was divided into two zones: center and periphery. The rats were released at the center of the field, where they were allowed to move freely for 2 min. Following every trial, the testing arena was thoroughly sanitized using ethanol (75%) to ensure complete elimination of any residual odors that might influence subsequent trials.

#### 2.8.3 Social interaction test (SIT)

Pairs of rats were placed in a box for 15 min to evaluate the changes in their social behavior. The assessment focused on the duration of interactions between rats, the types of social behaviors observed (e.g., sniffing, following), and exploratory behavior (File and Seth, 2003).

### 2.9 Animal euthanasia and tissue collection

On day 21 of treatment, all rats were euthanized using an intraperitoneal overdose of sodium pentobarbital (Eleiwa et al., 2024). The brain was rapidly removed, rinsed with ice-cold saline, and the two hemispheres were separated. One hemisphere was fixed in formalin (10%) for histological studies, while the cerebral cortex from the other hemisphere was dissected and divided into two parts: the first part was used for biochemical studies, and the second part was reserved for quantitative real-time PCR (qRT-PCR).

### 2.10 Homogenate preparation

The cerebral cortex tissue was homogenized (10% w/v) in ice-cold 0.1 M phosphate buffer (pH 7.4). The resulting homogenate was then centrifuged at 3,000 rpm for 15 min at 4 °C and the supernatant was stored at −20°C until further use (Tsakiris et al., 2004).

### 2.11 Assessment of free amino acids, monoamines and purinergic metabolites

High-performance liquid chromatography (HPLC) was used to measure the free amino acids levels [glutamate (Glu), aspartate (Asp), gamma-aminobutyric acid (GABA), and glycine (Gly)], monoamines levels [norepinephrine (NE), dopamine (DA), and serotonin (5-HT)], and purinergic metabolites in term of ATP in the rats’ cerebral cortex tissue.

Cerebral cortex tissue was homogenized in 70% HPLC-grade methanol, followed by centrifugation at 5,000 rpm for 10 min at 4 °C, and the resulting supernatant was used for analysis (El-bakry et al., 2024).

For monoamine analysis, HPLC (Agilent HP 1200 series, United States) with UV detection was used. Data acquisition and chromatogram analysis were performed using ChemStation program. Samples were separated on an AQUA C18 column (5 μm particle size, 150 × 4.6 mm; Phenomenex) with a mobile phase of 20 mM potassium phosphate (pH 2.5) and a flow rate of 1.5 mL/min, detected at 210 nm. Monoamines were quantified against Sigma-Aldrich standards (NE, DA, 5-HT) and expressed as μg/g tissue (Pagel et al., 2000; Abd-Elkareem et al., 2025).

Free amino acids were quantified using the pre-column phenylisothiocyanate (PITC) derivatization method described by (Heinrikson and Meredith, 1984), with HPLC separation on an Ultrasphere C18 column (5 μm particle size, 250 × 4.6 mm; Phenomenex) and UV detection at 254 nm. Amino acid calibration standards were obtained from Sigma-Aldrich (Saleh et al., 2024).

ATP content was analyzed using an Ultrasphere ODS C18 column (5 μm particle size, 250 × 4.6 mm; Analytics-Shop) with UV detection at 254 nm, using ATP standards from Sigma-Aldrich for calibration (Saleh et al., 2024).

The chromatogram and standard plots for the HPLC detection method for section 3.6. Changes in Free Amino Acids, Monoamines, and Purinergic Metabolites were provided in the supplementary data (Supplementary Figures S1-S9).

### 2.12 Assessment of BDNF and acetylcholinesterase (AChE) activity

Rat ELISA kits (Elabscience, Houston, TX, United States) were utilized to quantify the concentrations of BDNF (Cat. No.: E-EL-R1235) and AChE (Cat. No.: E-EL-R0355) in cerebral cortex tissue homogenate.

### 2.13 Histological examinations

The hemisphere tissues were fixed in formalin (10%) overnight to preserve tissues architecture and cellular morphology. The tissues were then dehydrated in alcohol, embedded in high-quality paraffin wax, and sectioned into approximately 4 µm thick slices. Serial sections underwent xylene deparaffinization and graded alcohol rehydration before being stained with hematoxylin and eosin (H&E) for routine histology. The sections were subsequently examined under a Nikon Eclipse E200-LED microscope (Tokyo, Japan) at ×200 and ×400 magnification to assess histopathological changes in the cerebral cortex (El-Marasy et al., 2021).

### 2.14 Assessment of oxidative stress and antioxidant parameters

Oxidative stress and antioxidant parameters were quantified in the cerebral cortex homogenate. Appropriate kits (Bio-Diagnostic Co., Egypt) were used for the determination of malondialdehyde (MDA), nitric oxide (NO), glutathione (GSH), glutathione peroxidase (GPx), superoxide dismutase (SOD), and catalase (CAT), spectrophotometrically.

### 2.15 Assessment of inflammatory parameters

The expression levels of inflammatory parameters in cerebral cortex tissue homogenate were quantified by rat ELISA kits (Novus Biologicals, Centennial, CO, United States) including NF-κB (Cat. No.: NBP3-42721), TNF-α (Cat. No.: NBP1-92681), IL-1β (Cat. No.: NBP1-92702), and IL-6 (Cat. No.: NBP1-92697).

### 2.16 Immunohistochemistry (IHC) examinations

IHC analysis for BDNF and GFAP was performed according to (Li Q. et al., 2015), with some modifications, to assess their distribution and expression in brain tissue using paraffin-embedded sections. The tissues were first dewaxed and rehydrated, followed by citrate buffer antigen retrieval (pH 6.0), and blocking of endogenous peroxidase activity with hydrogen peroxide (3%). Sections were incubated overnight at 4 °C with primary antibodies against BDNF and GFAP (1:500 dilution, Cat. No.: sc-546 and sc-6170, respectively, Santa Cruz Biotechnology, CA, United States) and then with goat anti-rabbit biotinylated secondary antibody (1:1000 dilution, Abcam ab6720) for 1 h at room temperature. The sections were then treated with an avidin-biotin horseradish peroxidase complex (HRP) and 3,3ʹ-diaminobenzidine (DAB) to visualize immunoreactivity. Counterstaining with hematoxylin was performed to highlight cellular structures, after which the sections were dehydrated, cleared in xylene, and mounted for imaging. The expression patterns of BDNF and GFAP were visualized using a Nikon Eclipse E200-LED microscope (Tokyo, Japan) at ×400 magnification.

### 2.17 Assessment of apoptotic activity

Apoptosis-related proteins were detected in cerebral cortex tissue homogenate using rat ELISA kits (Elabscience, Houston, TX, United States). The analysis included measurement of the anti-apoptotic protein BCL-2 (Cat. No.: E-EL-R0096), the pro-apoptotic protein BAX (Cat. No.: E-EL-R0098), and the caspase protein Caspase-3 (Cat. No.: E-EL-R0160).

### 2.18 Quantitative real-time PCR (qRT-PCR)

Total RNA was isolated from cerebral cortex tissue using TRIzol reagent (Invitrogen, Thermo Fisher Scientific). Subsequently, cDNA was obtained from the extracted RNA using a reverse transcription kit (Qiagen, Hilden, Germany). The qRT-PCR was conducted using the SYBR Green qPCR Master Mix kit (Qiagen, Hilden, Germany), and primers were obtained from Qiagen. The following genes were investigated for mRNA expression: BDNF, 5-HT_1A_, GFAP, Nrf2, NF-κB, and TNF-α, with glyceraldehyde-3-phosphate dehydrogenase (GAPDH) as the reference gene. Amplification and analysis were performed using an Applied Biosystems StepOne™ Real-Time PCR System with software version 3.1 (United States). Gene expression differences were evaluated using the 2^−ΔΔCT^ method (Albrakati et al., 2021). Primer sequences detailed in Table 1.

**TABLE 1:** Primer sequences corresponding to each gene.

Gene	Forward primer (5′-3′)	Reverse primer (5′-3′)
GAPDH	CAG​CCG​CAT​CTT​CTT​GTG​C	ATC​CGT​TCA​CAC​CGA​CCT​TC
BDNF	AGG​GAA​ATC​TCC​TGA​GCC​GA	TAA​TCC​AAT​TTG​CAC​GCC​GC
5-HT_1A_	GCT​CCT​TGG​CGG​TTA​CTG​AT	GGT​CCA​CTT​GTT​GAG​CAC​CT
GFAP	GTT​ACC​AGG​AGG​CAC​TTG​CT	TGG​CAG​GGC​TCC​ATT​TCA​A
Nrf2	GGC​TGG​GAA​TAT​CCA​GGG​C	GGT​TGC​CCA​CAT​TCC​CAA​AC
NF-κB	TCC​TTT​CGG​AAC​TGG​GCA​AA	AGG​TAT​GGG​CCA​TCT​GTT​GAC
TNF-α	ACT​GAA​CTT​CGG​GGT​GAT​CG	GCT​TGG​TGG​TTT​GCT​ACG​AC

### 2.19 Statistical analysis

Statistical analyses were performed using GraphPad Prism software (version 9.0, GraphPad Software, Inc., La Jolla, CA), with data presented as mean ± standard error of the mean (SEM). For multiple comparisons, one-way analysis of variance (ANOVA) was used, followed by Tukey’s *post hoc* test. Two-sample comparisons were conducted using the independent samples t-test. Statistical significance was considered at P < 0.05. All statistical figures were generated using GraphPad Prism software.

## 3 Results

### 3.1 Total phenolics and flavonoid content analysis

The total phenolics content in the leek extract was quantified as 625.17 mg GAE/g extract. Additionally, the flavonoid content was found to be 112.2 mg QE/g extract. These values indicate that the leek extract is rich in both phenolics and flavonoids.

### 3.2 FT-IR analysis

**FIGURE 2:**
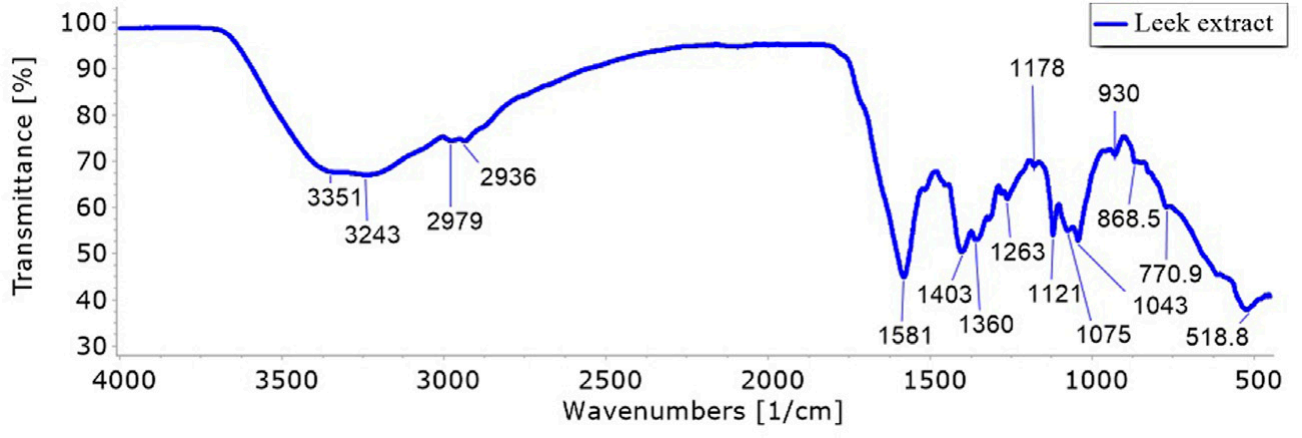
FT-IR spectra of the phytochemical compounds in *Allium ampeloprasum* var. *Kurrat* leaves extract.

The FT-IR analysis of Egyptian leek revealed several distinct functional groups, aiding in the identification of its chemical composition. The strong, broad peak at 3,239.04 cm^-1^ indicates O-H stretching and confirms the presence of carboxylic acid groups with significant hydrogen bonding. The medium peak at 1581.99 cm^-1^ is attributed to C=C stretching, suggesting the existence of cyclic alkenes (Figure 2). The strong peak at 1403.34 cm^-1^, associated with O-H bending, further supports the presence of carboxylic acids, while the medium peak at 1360.7 cm^-1^ corresponds to O-H bending in alcohols. The strong peak at 1262.72 cm^-1^ is due to C-O stretching in aromatic esters, and another strong peak at 1120.71 cm^-1^ is assigned to C-O stretching in aliphatic ethers. Additionally, the strong peak at 1043.2 cm^-1^ signifies S=O stretching in sulfoxides, and the peak at 519 cm^-1^ is attributed to C-X stretching in halo compounds. This analysis reveals a complex mixture of functional groups in Egyptian leek, including carboxylic acids, cyclic alkenes, alcohols, esters, ethers, sulfoxides, and halo compounds, reflecting its diverse chemical composition. The strong and broad O-H stretching peak is particularly notable, indicating significant hydrogen bonding, which is typical for plant materials.

### 3.3 LC-MS analysis

**FIGURE 3:**
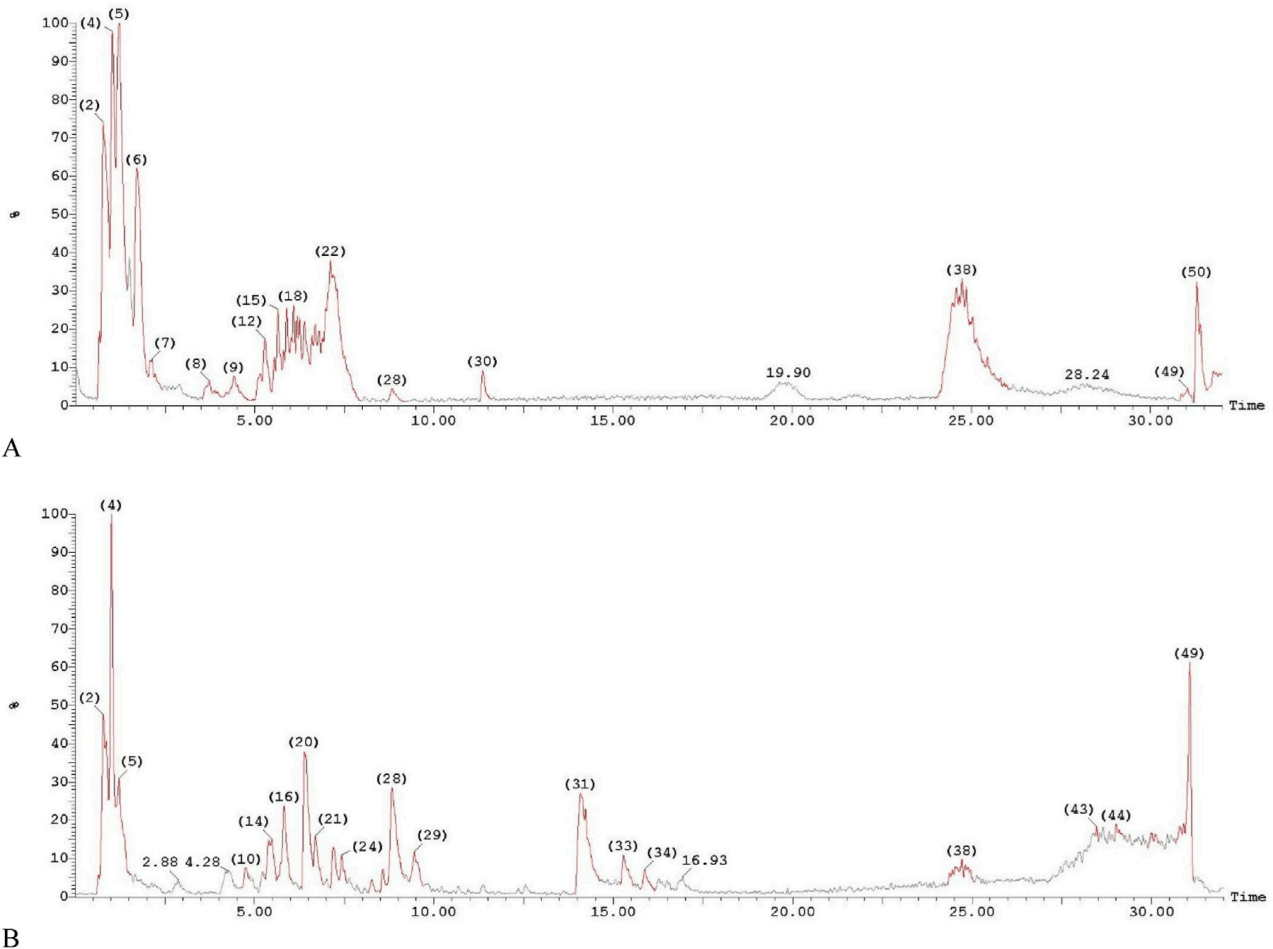
LC-MS/MS chromatograms of the phytochemical compounds in *Allium ampeloprasum* var. *Kurrat* leaves extract. **(A)** Positive ion acquisition mode, and **(B)** Negative ion acquisition mode.

The LC-MS analysis of the Egyptian leek extract leaves revealed the presence of 26 distinct compounds (after eliminating unknown compounds), based on their unique fragmentation patterns, underscoring the extract’s rich bioactive profile (Figure 3). The analysis demonstrated that the leek extract is abundant in phytochemicals, with sulfur-containing compounds predominating, aligning with previous reports of sulfur’s bioactive properties in Allium species (Poojary et al., 2017). The phytochemicals were listed according to their retention time (Rt), as shown in Table 2.

**TABLE 2:** Identification of phytochemical compounds by LC-MS in Allium ampeloprasum var. Kurrat leaves extract.

Compound	RT	MW (g/mol)	Ionization mode	Area %total
S-Methyl-L-cysteine	1.01	135.19	[M + H]+	8.48
Dipropyl disulfide	1.01	150.3	[M – H]−	15.63
Phenylalanine	1.21	165.19	[M + H]+	11.15
Arabinofuranose	1.21	150.13	[M – H]−	7.66
Tryptophan	1.71	204.23	[M + H]+	6.62
Quercetin	2.12	302.23	[M + H]+	0.70
Apigenin 7-O-glucoside	3.74	432.4	[M + H]+	1.01
Trans-Zeatin-O-glucoside riboside	5.39	513.5	[M – H]−	1.51
Quercetin-7-O-rutinoside	5.83	610.5	[M – H]−	4.49
Kaempferol 3-glucoside	6.39	448.38	[M + H]+	2.67
Beta-Carotene	6.69	536.9	[M – H]−	2.71
Gallic Acid	7.20	170.12	[M – H]−	2.02
Quercetin 3-O-rhamnoside	8.27	448.4	[M – H]−	0.48
Luteolin 7-O-glucoside	8.57	448.4	[M – H]−	0.84
Cyclic adenosine monophosphate (cAMP)	8.84	329.205	[M – H]−	7.65
Orotidylic acid (Orotidine 5′-monophosphate)	9.45	368.19	[M – H]−	2.19
Chlorogenic acid	15.87	354.31	[M – H]−	1.23
Coumarin	28.48	146.14	[M – H]−	0.23
Succinic acid	29.02	118.09	[M – H]−	0.87
S-Methylcysteine sulfoxide	29.99	151.19	[M – H]−	0.52
L-valine	30.09	117.15	[M – H]−	0.41
Allicin	30.78	162.28	[M – H]−	1.03
L-Cysteine	31.04	121.16	[M + H]+	0.38
Diallyl disulfide	31.04	146.3	[M – H]−	8.18
L-Isoleucine	31.30	131.17	[M + H]+	3.75

### 3.4 Molecular docking analysis and evaluation of the leek extract

**FIGURE 4:**
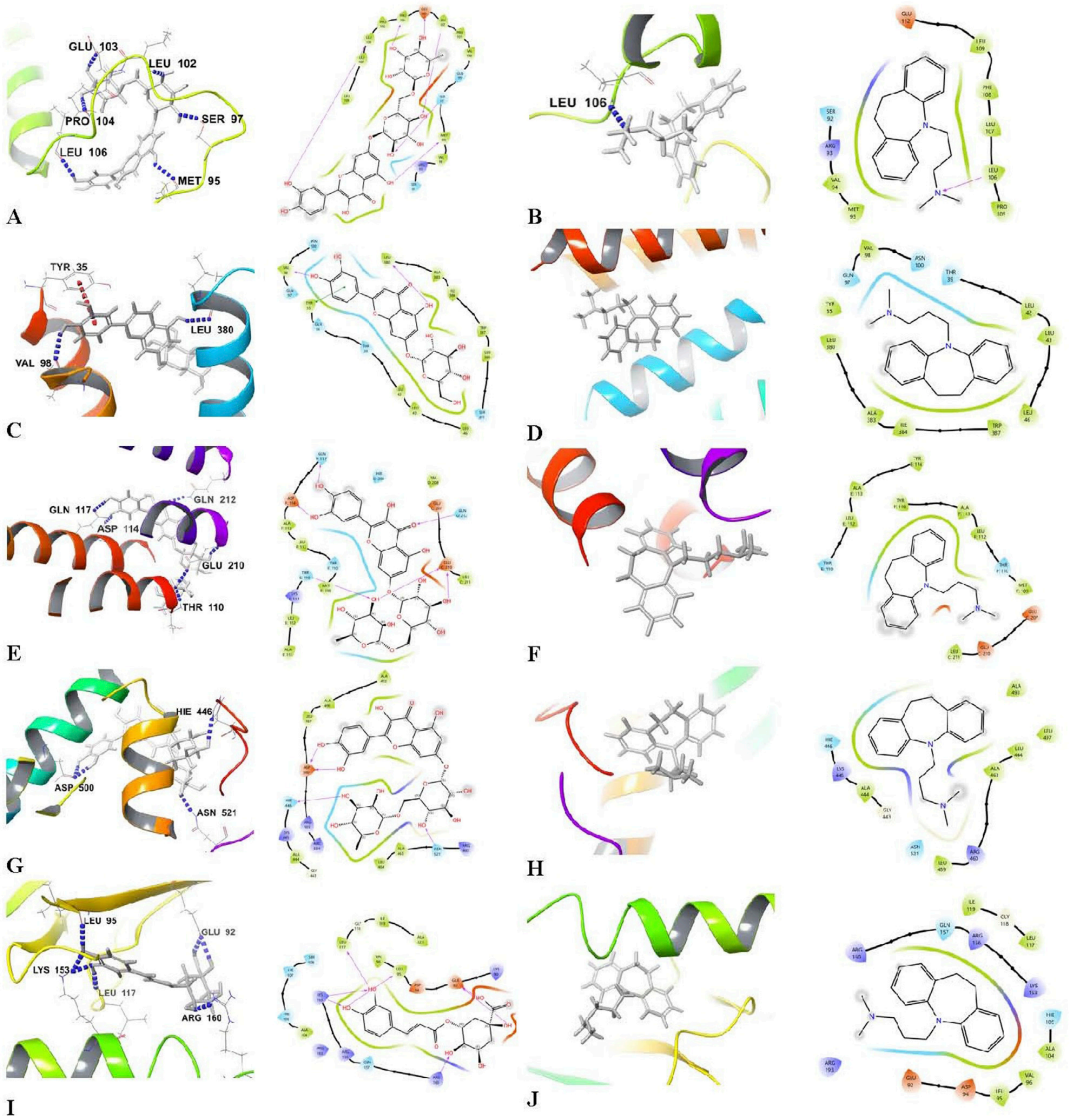
3D molecular docking representations and 2D schematic view of docking Interactions of bioactive components in Egyptian leek and target proteins. **(A)** Quercetin-7-O-rutinoside-BDNF, **(B)** Imipramine-BDNF, **(C)** Luteolin 7-O-glucoside-5-HT_1A_, **(D)** Imipramine-5-HT_1A_, **(E)** Quercetin-7-O-rutinoside-GFAP, **(F)** Imipramine-GFAP, **(G)** Quercetin-7-O-rutinoside-Nrf2, **(H)** Imipramine-Nrf2, **(I)** Chlorogenic acid-NF-κB, and **(J)** Imipramine-NF-κB.

Molecular docking studies on the bioactive components in Egyptian leek demonstrated robust binding affinities and extensive hydrogen bonding interactions with key proteins implicated in depression and its related pathways. These proteins include BDNF, 5-HT_1A_, GFAP, Nrf2, and NF-κB (Tables 3, 4; Figure 4). Lower (more negative) docking scores indicate stronger binding interactions, and the analysis highlighted flavonoids as the most promising compounds. Quercetin-7-O-rutinoside had the highest affinity for BDNF with a docking score of −9.946 kcal/mol, forming six hydrogen bonds with the residues MET 95, SER 97, LEU 102, GLU 103, PRO 104, and LEU 106. For the 5-HT_1A_, Luteolin 7-O-glucoside recorded the highest binding affinity (−8.237 kcal/mol), and interacted with the receptor through two hydrogen bonds involving VAL 98 and LEU 380. Quercetin-7-O-rutinoside again exhibited stronger binding to GFAP (−8.244 kcal/mol; six hydrogen bonds) and Nrf2 (−7.032 kcal/mol; four hydrogen bonds). Other flavonoids, such as quercetin, kaempferol 3-glucoside, and apigenin 7-O-glucoside, demonstrated strong binding across multiple targets, with extensive hydrogen bonding to critical residues (for example, quercetin with MET 95, LEU 106, LEU 107 in BDNF). Chlorogenic acid emerged as the strongest binder to NF-κB, with a docking score of −7.845 kcal/mol and formation of seven hydrogen bonds with residues GLU 92, LEU 95, LEU 117, LYS 153, and ARG 160. In contrast, sulfur-containing compounds such as diallyl disulfide, dipropyl disulfide and allicin showed weak binding affinities (with scores near zero or only slightly negative) and did not form any hydrogen bonds, except for allicin, which formed a single hydrogen bond with each target protein. Imipramine showed moderate binding affinities ranging from −1.923 to −7.048 kcal/mol and primarily formed limited hydrogen bonds with BDNF (notably with LEU 106). Notably, the flavonoids derived from leek outperformed imipramine in both binding strength and interaction complexity.

**TABLE 3:** The docking score values for representative compounds with target proteins.

Docking score (kcal/mol)		BDNF	5-HT_1A_	GFAP	Nrf2	NF-κB
Bioactive components in Egyptian leek	Quercetin	−6.848	−7.351	−4.639	−5.476	−5.790
	Quercetin-7-O-rutinoside	−9.946	−7.620	−8.244	−7.032	−5.718
	Kaempferol 3-glucoside	−7.654	−6.904	−3.076	−5.568	−6.271
	Luteolin 7-O-glucoside	−8.234	−8.237	−5.909	−3.061	−4.236
	Apigenin 7-O-glucoside	−6.386	−7.189	−4.181	−3.019	−5.812
	Phenylalanine	−3.627	−3.538	−1.890	−2.284	−3.973
	Tryptophan	−4.057	−5.087	−2.712	−2.883	−4.784
	L-Isoleucine	−2.981	−3.348	−2.030	−3.458	−4.337
	cAMP	−5.159	−4.450	−4.049	−4.187	−4.380
	Gallic acid	−5.816	−5.377	−4.941	−6.011	−6.692
	Chlorogenic acid	−7.609	−7.129	−1.449	−4.378	−7.845
	S-Methyl-L-cysteine	−3.001	−2.554	−1.459	−3.772	−3.809
	Dipropyl disulfide	−1.384	−2.404	−1.792	−1.011	−2.059
	Diallyl disulfide	−0.941	−1.522	−1.050	−1.090	−1.124
	Allicin	−2.315	−2.520	−2.285	−2.052	−2.568
Drug	Imipramine	−3.347	−4.784	−1.923	−3.560	−2.791

**TABLE 4:** The hydrogen (H) bonding interactions between representative compounds and amino acid residues of target proteins.

H- bond interactions		BDNF	5-HT_1A_	GFAP	Nrf2	NF-κB
Bioactive components in Egyptian leek	Quercetin	MET 95, LEU 106, LEU 107	VAL 98, ASN 100	C:GLU 210, D:GLN 212, F:ASP 114, F:GLN 117	LEU 454, ASN 521	GLU 92, LEU 117, LYS 153, GLN 157, ARG 160
	Quercetin-7-O-rutinoside	MET 95, SER 97, LEU 102, GLU 103, PRO 104, LEU 106	ASN 100	C:GLU 210, D:GLN 212, E:THR 110, F:ASP 114, F:GLN 117	HIE 446, ASP 500, ASN 521	GLU 92, ASP 94, ARG 160, GLU 177
	Kaempferol 3-glucoside	MET 95, LEU 107, GLU 112	VAL 98, ASN 100	C:GLU 207, C:GLU 210	GLY 443, HIE 446, ASP 457, ARG 460, ASP 500, ARG 504, LYS 518	LYS 90, GLU 92, ASP 94, LEU 117, ARG 160
	Luteolin 7-O-glucoside	MET 95, LEU 106, LEU 107, LEU 109, GLU 112	VAL 98, LEU 380	C:GLU 207, C:GLU 210, D:ARG 201, D:GLU 205, E:TYR 116, F:GLN 117	HIE 446, ASP 457, ARG 504	SER 115, ARG 156
	Apigenin 7-O-glucoside	SER 92, MET 95, GLU 103, LEU 106	ASN 100	C:GLU 207, C:GLU 210, E:TYR 116, F:GLN 117	GLY 443, LYS 518	LYS 90, GLU 92, LEU 117, LYS 153, ARG 160
	Phenylalanine	LEU 106	GLN 97, ASN 100	C:GLU 210	GLY 443, HIE 446	LEU 117
	Tryptophan	SER 92	THR 39, ASN 100	C:GLU 210, E:THR 110	HIE 446	LEU 95, GLY 118
	L-Isoleucine	ARG 93, LEU 106	THR 39, GLN 97	C:GLU 210	GLY 443, HIE 446	LEU 117, LYS 153
	cAMP	ARG 93, MET 95, SER 97	TYR 35, ASN 100	C:GLU 210, F:GLN 117	ALA 444, ASN 521	GLU 92, LYS 153, SER 195
	Gallic acid	SER 92, LEU 107	ASN 100	C:GLU 207, C:GLU 210, E:TYR 116, F:THR 110	ALA 444, ASN 521	LEU 95, LEU 117, LYS 153, GLN 157, ARG 193
	Chlorogenic acid	SER 92, MET 95, SER 97, LEU 106	VAL 98, ASN 100	C:GLU 207, C:GLU 210, E:THR 110	GLY 443, ASP 457, ARG 504	GLU 92, LEU 95, LEU 117, LYS 153, ARG 160
	S-Methyl-L-cysteine	LEU 106, LEU 107	TYR 35, ASN 100	C:GLU 210, E:THR 110	GLY 443, HIE 446	LEU 117, LYS 153
	Dipropyl disulfide	-	-	-	-	-
	Diallyl disulfide	-	-	-	-	-
	Allicin	MET 95	TYR 35	E:THR 110	ALA 444	LYS 153
Drug	Imipramine	LEU 106	-	-	-	-

### 3.5 Body weight and behavioral assessments

**FIGURE 5:**
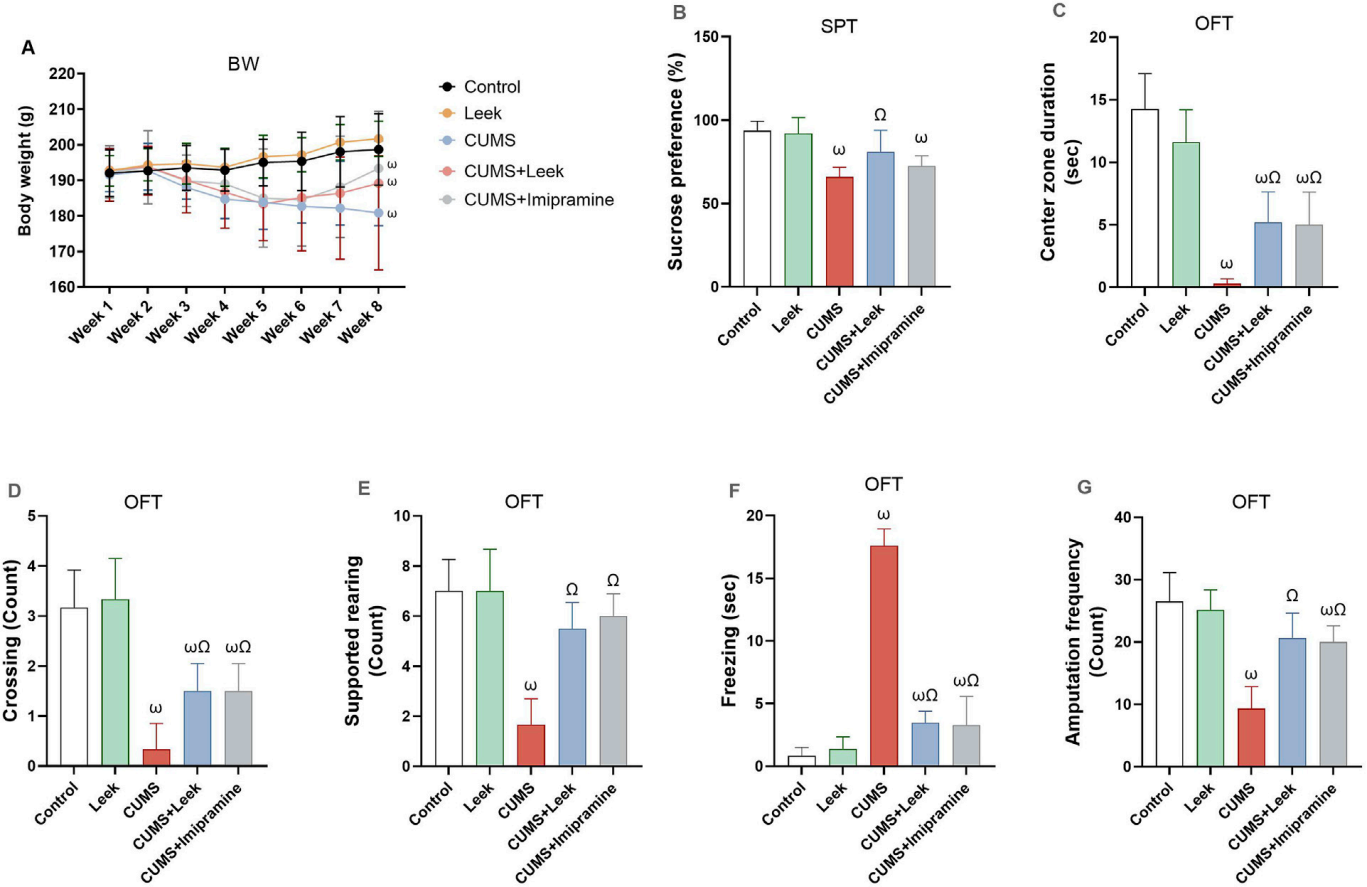
Body weight and behavioral assessments performed to evaluate the effects of CUMS exposure and leek treatment. **(A)** BW over 8 weeks, **(B)** SPT, **(C)** Center zone duration (sec) in the OFT. **(D)** Crossing in the OFT, **(E)** Supported rearing in the OFT, **(F)** Freezing (sec) in the OFT, and **(G)** Amputation frequency (squares crosses) in the OFT. Data are expressed as mean ± SEM (n = 6 rats/group). For statistical significance, ωP < 0.05, compared to Control group; ΩP < 0.05, compared to CUMS group (one-way ANOVA followed by Tukey’s test).

Changes in the body weight across the different groups of rats over the 8-week period were shown in Figure 5A. While no significant change in body weight was observed in the Control and Leek groups, all CUMS groups exhibited a significant reduction in weight after 4 weeks of CUMS induction in comparison to the Control group (ωP < 0.001). By the end of the experiment, all groups exhibited no significant increase in body weight.

CUMS exposure is known to suppress the brain reward system (Albrakati et al., 2021), as evidenced by the reduced sucrose consumption in the rats exposed to CUMS, in comparison to the Control group (ωP < 0.001). Treatment with leek, however, significantly increased sucrose preference, in comparison to the CUMS group (ΩP < 0.05), while treatment with imipramine did not result in a significant change (Figure 5B).

In addition to changes in sucrose preference, CUMS exposure was associated with alterations in exploratory behaviors and heightened fear responses (Figures 5C–G). Specifically, rats exposed to CUMS spent less time in the center zone of the open field arena, crossed fewer squares, and exhibited reduced supported rearing and locomotor activity (ωP < 0.001). These rats also displayed a significant increase in freezing behavior (ωP < 0.001), in comparison to the Control group, indicating heightened anxiety and reduced exploratory activity. Importantly, treatment with leek or imipramine significantly reversed these behavioral deficits by reducing the freezing behavior (ΩP < 0.001) and restoring the time spent in the center zone (ΩP < 0.05), the number of crossings (ΩP < 0.05), and the frequency of supported rearing and amputation behaviors (ΩP < 0.001), in comparison to the CUMS group.

SIT was also performed as part of the behavioral assessment on both CUMS-exposed and control rats, to assess the impact of CUMS on social behaviors. The data were observational and qualitatively analyzed (e.g., interaction and avoidance behaviors). CUMS-exposed rats predominantly exhibited reduced social interaction and were characterized by social avoidance. These rats spent significantly less time near or engaging in social behaviors with other rats, with some displaying increased aggression or fighting. Moreover, these CUMS-exposed rats tended to remain in the corners of the arena, suggesting impaired social motivation or increased anxiety. In contrast, control rats displayed markedly different behavior in the SIT, engaging more frequently in social interactions such as sniffing and following one another, and spending more time in social proximity. This reflects typical social behavior and suggests normal social motivation. Control rats also exhibited balanced exploratory behavior within the test area. Collectively, these observations emphasize the impact of CUMS on social engagement and highlight the behavioral differences between stressed and non-stressed rats. Notably, both leek and imipramine treatment led to significant improvements in social interaction in the CUMS-exposed rats.

### 3.6 Changes in Free Amino Acids, monoamines, and purinergic metabolites

**FIGURE 6:**
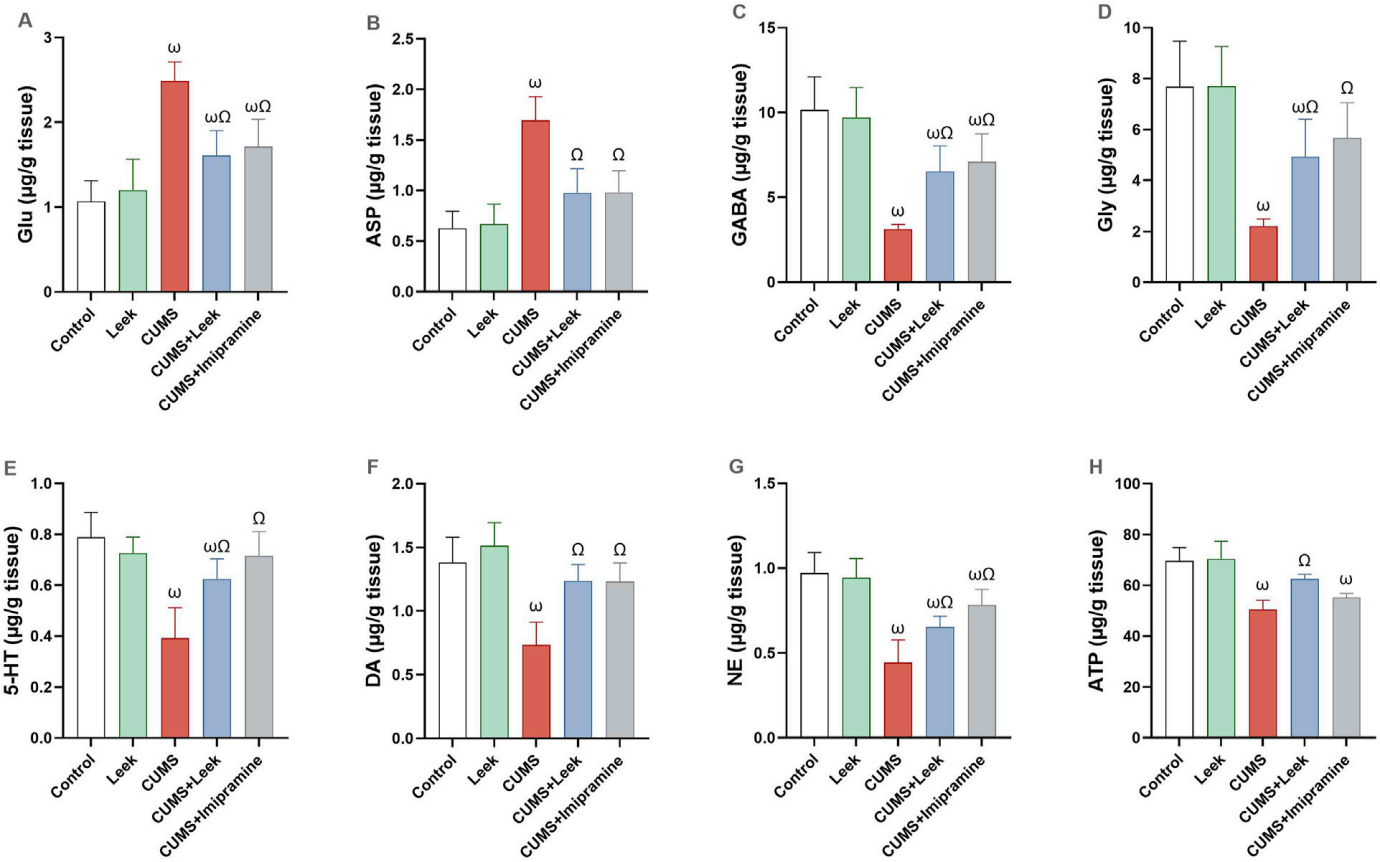
Neurochemical changes in the cerebral cortex of rats exposed to CUMS and the effects of leek treatment. **(A)** Glu, **(B)** Asp, **(C)** GABA, **(D)** Gly, **(E)** 5-HT, **(F)** DA, **(G)** NE, and **(H)** ATP levels following CUMS exposure and leek treatment. Data are expressed as mean ± SEM (n = 6 rats/group). For statistical significance, ωP < 0.05, compared to Control group; ΩP < 0.05, compared to CUMS group (one-way ANOVA followed by Tukey’s test).

A disturbance in the levels of free amino acids, monoamines, and purinergic metabolites was observed in the cerebral cortex of rats exposed to CUMS. Specifically, levels of Glu and Asp were significantly elevated (both ωP < 0.001), whereas GABA, Gly, 5-HT, DA, NE, and ATP were significantly decreased (all ωP < 0.001), in comparison to the Control group. Notably, leek administration demonstrated a potent neuromodulatory effect, as it significantly restored the levels of neurotransmitters 5-HT, DA, NE (ΩP < 0.01, 0.001, 0.05 respectively), along with GABA, Gly, and ATP (ΩP < 0.01, 0.05, and 0.001, respectively), and concurrently reducing the levels of Glu and Asp (both ΩP < 0.001), in comparison to the CUMS group. Similarly, imipramine treatment normalized the levels of the monoamines (all ΩP < 0.001) and amino acids (all ΩP < 0.01), although no significant changes in ATP levels were observed in the imipramine-treated rats (ΩP > 0.05) (Figure 6).

### 3.7 Changes in BDNF and AChE activity

**FIGURE 7:**
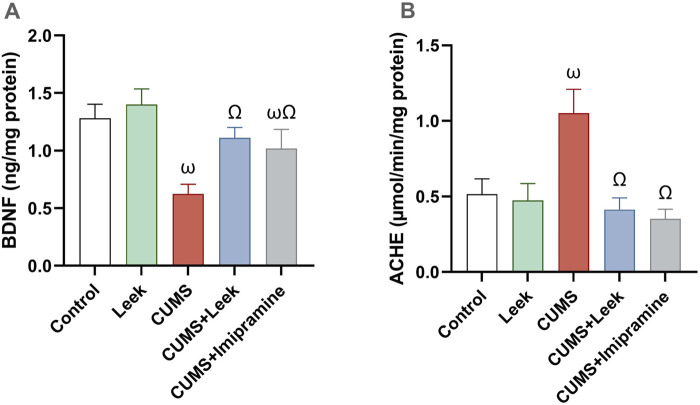
Changes in BDNF levels and AChE activity in the cerebral cortex of rats exposed to CUMS and the effects of leek treatment. **(A)** BDNF levels, and **(B)** AChE activity following CUMS exposure and leek treatment. Data are expressed as mean ± SEM (n = 6 rats/group). For statistical significance, ωP < 0.05, compared to Control group; ΩP < 0.05, compared to CUMS group (one-way ANOVA followed by Tukey’s test).

CUMS exposure was found to alter synaptic plasticity in several brain regions, including the cerebral cortex (Li X. L. et al., 2015). In CUMS-exposed rats, impairment of both neuroplasticity and cholinergic neurotransmission were observed (Figures 7A,B, respectively), as indicated by decreased BDNF levels and elevated AChE activity in the cortical tissue (both ωP < 0.001), in comparison to the Control group. Interestingly, treatment with leek significantly increased BDNF levels and reduced AChE activity in the cerebral cortex to near-normal values (both ΩP < 0.001), in comparison to the CUMS group. Imipramine administration was also found to normalize the changes in both BDNF and AChE levels (both ΩP < 0.001).

### 3.8 Histopathological changes of the cerebral cortex tissue

**FIGURE 8:**
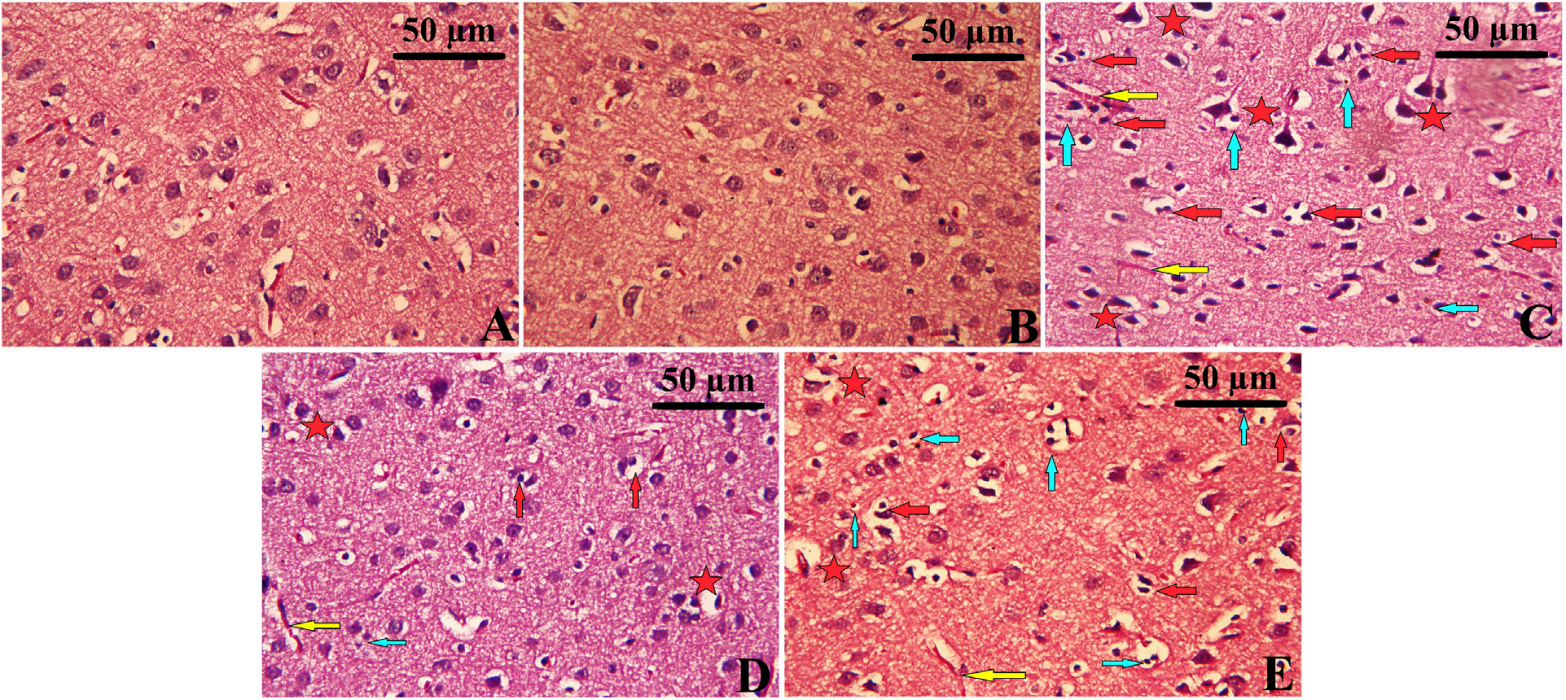
Histopathological changes observed in the cerebral cortex following CUMS induction and the effects of leek treatment. The cortical tissue of **(A)** Control, **(B)** Leek, **(C)** CUMS, **(D)** CUMS + Leek, and **(E)** CUMS + Imipramine groups. Red arrows indicate apoptotic neurons; yellow arrows indicate congested blood vessels; blue arrows indicate inflammatory cells infiltration; red stars indicate degenerative and edema neurons.

Histopathological analysis of the cerebral cortex revealed significant changes in the CUMS group (Figure 8C), characterized by severe neuronal degeneration. Cortical tissue from the CUMS group exhibited prominent features of neuronal damage, including an increased number of pyknotic neurons, nuclear condensation, development of vacuoles within the intraneuronal space, and infiltration of inflammatory cells. Neurons were sparsely arranged, with the cell boundaries becoming unclear, and some showing signs of necrosis and interstitial edema. In contrast, cortical tissue from the Control and Leek groups demonstrated intact architecture, with neurons arranged orderly, clear boundaries between nuclei and cytoplasm, and no significant inflammation (Figures 8A,B). The CUMS + Leek group showed a remarkable improvement in cortical structures, with a reduction in pyknotic neurons and neuronal shrinkage (Figures 8D,E). Notably, after treatment with imipramine, some neurons showed mild nuclear pyknosis and degeneration, while others remained intact, suggesting partial recovery. Additionally, H&E staining highlighted a reduction in neuronal gaps and restored cytoplasmic morphology following leek or imipramine treatment.

### 3.9 Changes in oxidative stress and antioxidant parameters

**FIGURE 9:**
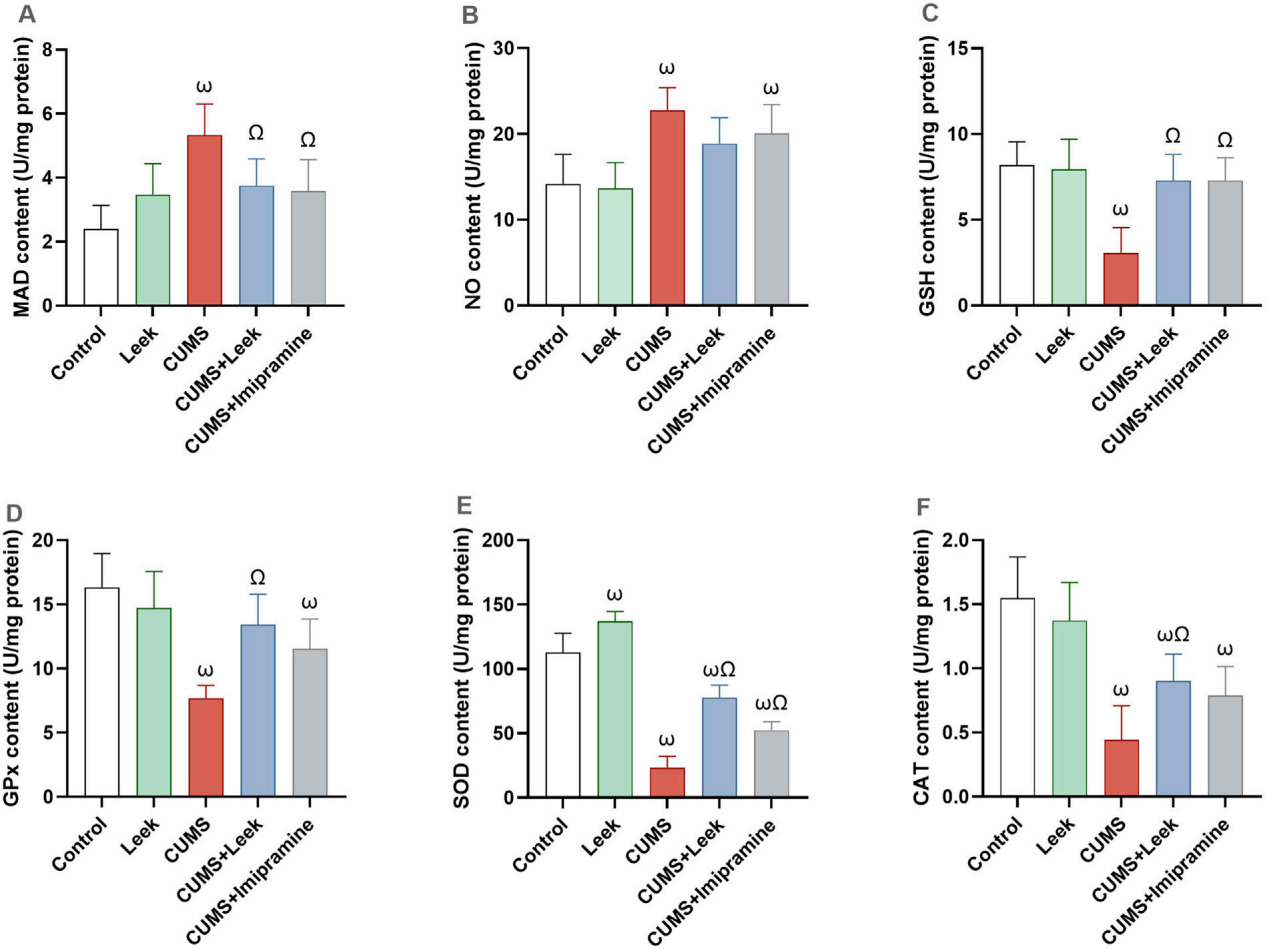
Oxidative stress and antioxidant defense in the cerebral cortex of rats exposed to CUMS and the effects of leek treatment. **(A)** MDA content, **(B)** NO content, **(C)** GSH content, **(D)** GPx content, **(E)** SOD content, and **(F)** CAT content. Data are expressed as mean ± SEM (n = 6 rats/group). For statistical significance, ωP < 0.05, compared to Control group; ΩP < 0.05, compared to CUMS group (one-way ANOVA followed by Tukey’s test).

CUMS has detrimental effects on the behavior and mood of rats. Exposure to CUMS resulted in significant oxidative damage in cortical tissue, evidenced by increased levels of MDA and NO (both ωP < 0.001), in comparison to the Control group. This oxidative stress was accompanied by reduction in endogenous antioxidant defense mechanisms, including GSH, and its associated enzyme GPx, SOD, and CAT (all ωP < 0.001), in comparison to the Control group. Treatment with leek effectively counteracted these oxidative disturbances, significantly reducing brain MDA levels (ΩP < 0.05), with only a slight effect on NO levels (ΩP > 0.05). It also restored antioxidant protein levels, including GSH, GPx, SOD, and CAT in the cortex (ΩP < 0.001, 0.01, 0.001, and 0.05, respectively), in comparison to the CUMS group. Imipramine demonstrated slight protective effects by decreasing MDA levels (ΩP < 0.05) with no significant effect on NO levels (ΩP > 0.05). It significantly increased brain GSH and SOD (ΩP < 0.001, and 0.01, respectively), and restored GPx and CAT levels (both ΩP > 0.05), although not significant (Figure 9).

### 3.10 Changes in inflammatory parameters

**FIGURE 10:**
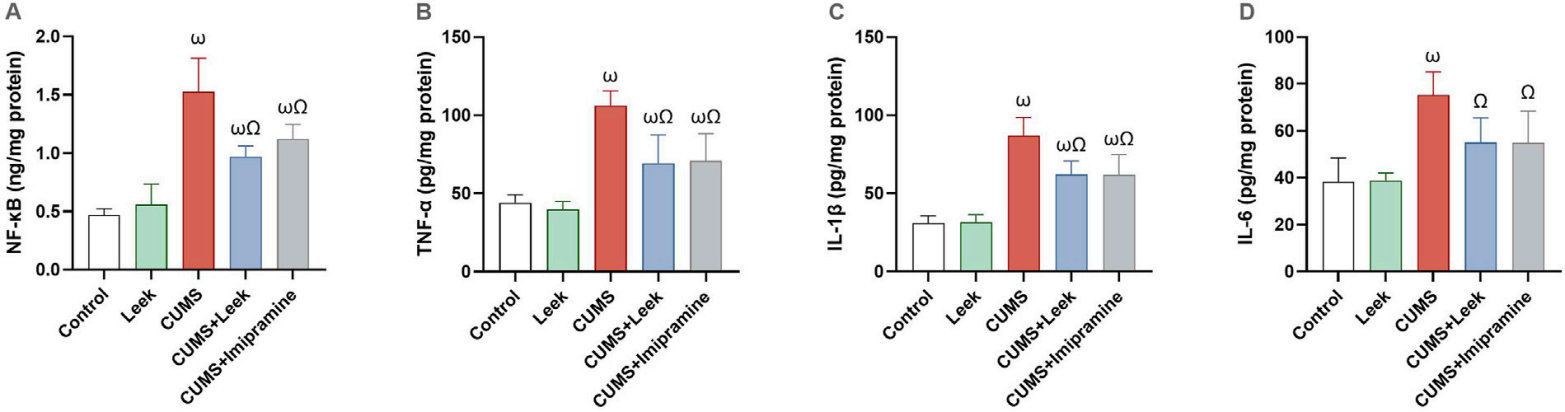
Changes in the inflammatory parameters in the cerebral cortex of rats exposed to CUMS and the effects of leek treatment. **(A)** NF-κB, **(B)** TNF-α, **(C)** IL-1β, and **(D)** IL-6 following CUMS exposure and leek treatment. Data are expressed as mean ± SEM (n = 6 rats/group). For statistical significance, ωP < 0.05, compared to Control group; ΩP < 0.05, compared to CUMS group (one-way ANOVA followed by Tukey’s test).

CUMS induced neuroinflammation, leading to the activation of pro-inflammatory factors that impair neuronal function (Zhang et al., 2019). CUMS exposure resulted in a significant elevation in the NF-κB, TNF-α, IL-1β, and IL-6 levels in the cortical tissue (all ωP < 0.001), in comparison to the Control group. Notably, leek treatment significantly modulated the inflammatory excesses induced by CUMS and ameliorated the elevations in NF-κB, TNF-α, IL-1β, and IL-6 levels (ΩP < 0.001, 0.001, 0.001, and 0.05, respectively), in comparison to the CUMS group. Similarly, imipramine treatment produced comparable anti-inflammatory effects, significantly reducing NF-κB (ΩP < 0.01), TNF-α, and IL-1β (ΩP < 0.001), although its impact on IL-6 was less pronounced (ΩP < 0.05) (Figure 10).

### 3.11 Immunohistochemistry analysis

**FIGURE 11:**
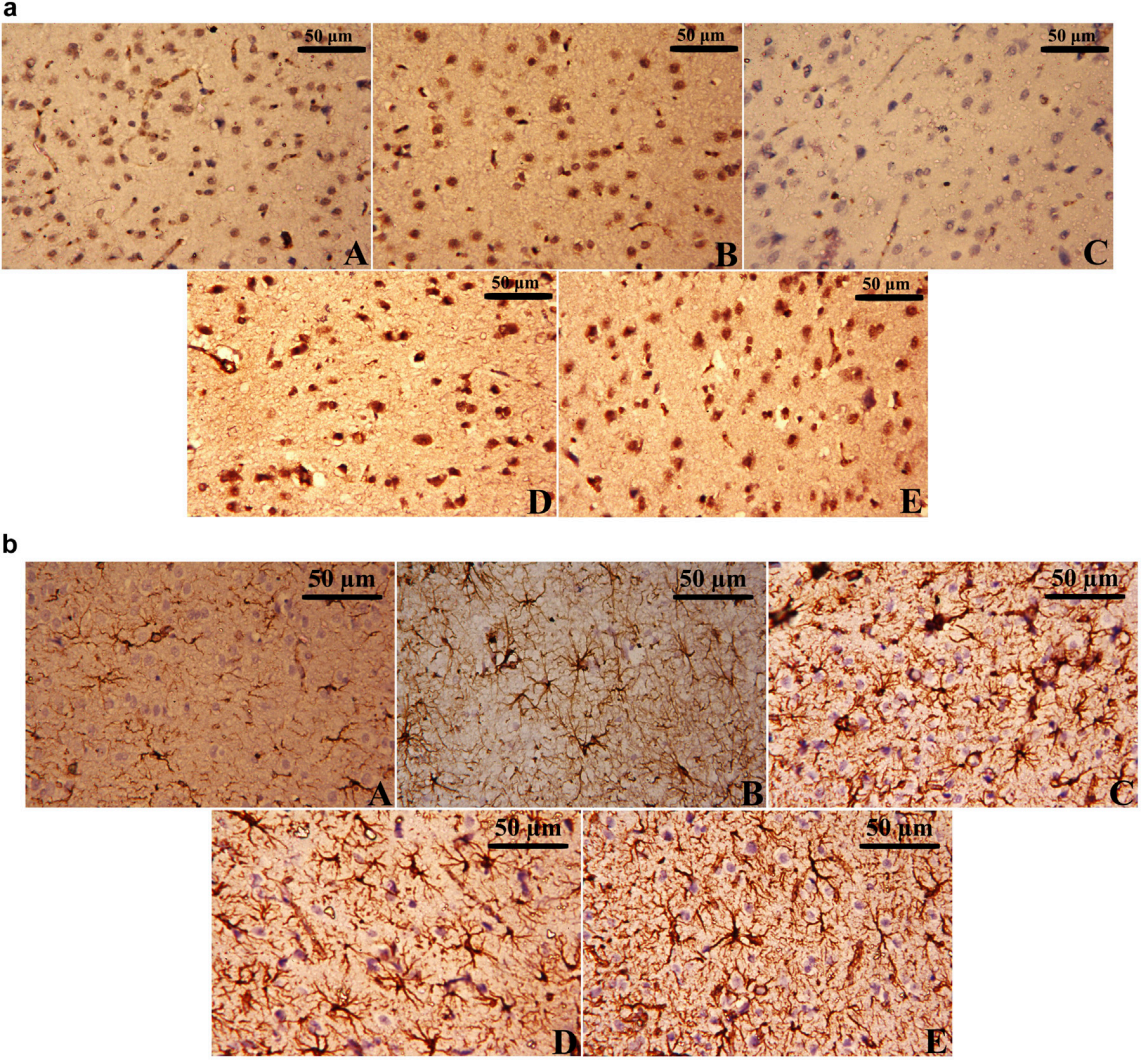
Immunohistochemical analysis of **(a)** BDNF-positive cells and **(b)** GFAP-positive cells in the cerebral cortex of CUMS-exposed rats and the effects of leek treatment. The cortical tissue of **(A)** Control, **(B)** Leek, **(C)** CUMS, **(D)** CUMS + Leek, and **(E)** CUMS + Imipramine groups.

CUMS exposure resulted in a marked reduction in BDNF-positive cells and an increase in GFAP-positive cells in the cerebral cortex (Figure 11C), reflecting both neuronal and glial dysfunction. In contrast, the Control and the Leek groups exhibited higher BDNF immunoreactivity and fewer GFAP-positive astrocytes, indicative of normal neurotrophic support and minimal astrocytic activation (Figures 11A,B). Notably, treatment with either leek or imipramine effectively restored BDNF-positive cells and decreased GFAP-positive cells in the cortical tissue of CUMS-exposed rats, in comparison to the CUMS group (Figures 11D,E).

### 3.12 Changes in apoptotic parameters

**FIGURE 12:**
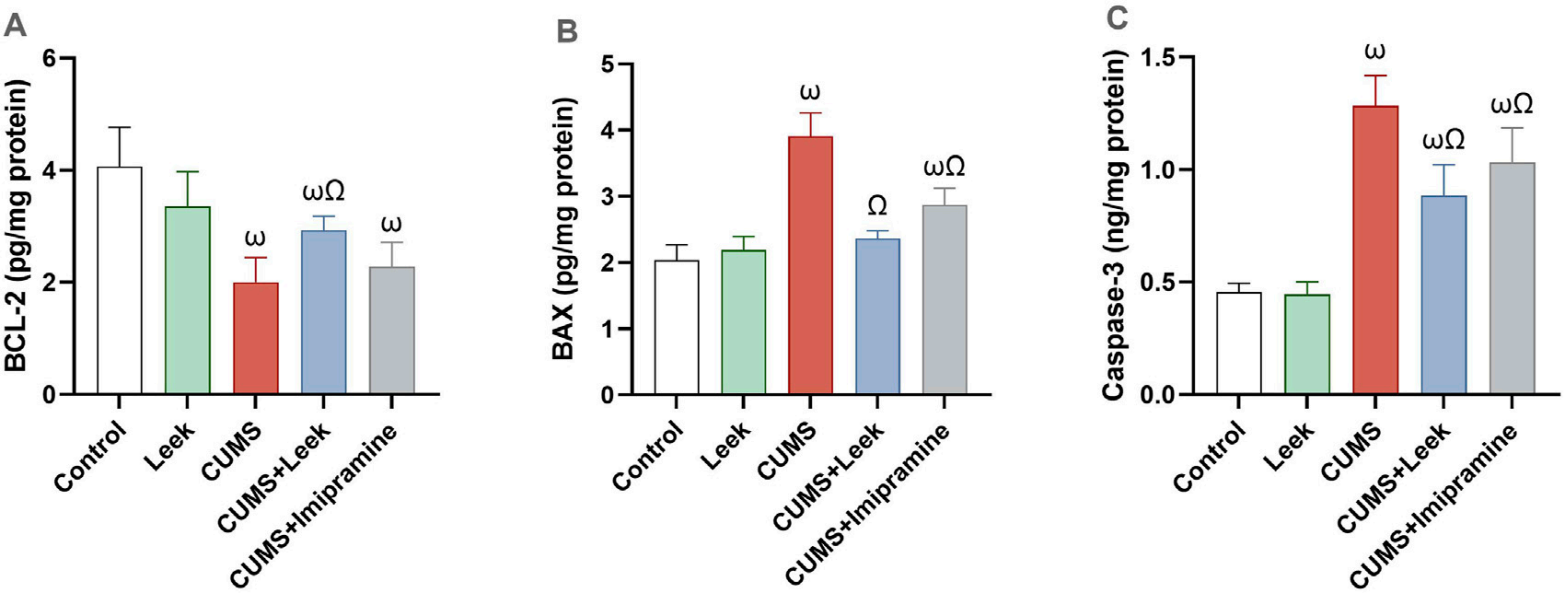
Changes in the anti-apoptotic and pro-apoptotic proteins in the cerebral cortex of rats exposed to CUMS and the effects of leek treatment. **(A)** BCL-2, **(B)** BAX, and **(C)** caspase-3 following CUMS exposure and leek treatment. Data are expressed as mean ± SEM (n = 6 rats/group). For statistical significance, ωP < 0.05, compared to Control group; ΩP < 0.05, compared to CUMS group (one-way ANOVA followed by Tukey’s test).

CUMS exposure leads to neuronal deterioration and apoptosis (Fan et al., 2019). The CUMS-exposed rats exhibited significant reduction in the levels of anti-apoptotic protein BCL-2 while elevation in the levels of pro-apoptotic proteins BAX and the caspase-3 (all ωP < 0.001), in comparison to the Control group. Treatment with leek restored the balance of these apoptosis-related proteins, elevating BCL-2 levels (ΩP < 0.05) while reducing BAX and caspase-3 levels (both ΩP < 0.001), in comparison to the CUMS group. In contrast, imipramine did not significantly alter BCL-2 levels (ΩP > 0.05). However, it significantly reduced the level of BAX and caspase-3 levels (ΩP < 0.001, and 0.01, respectively), in comparison to the CUMS group (Figure 12).

### 3.13 Gene expression analysis

**FIGURE 13:**
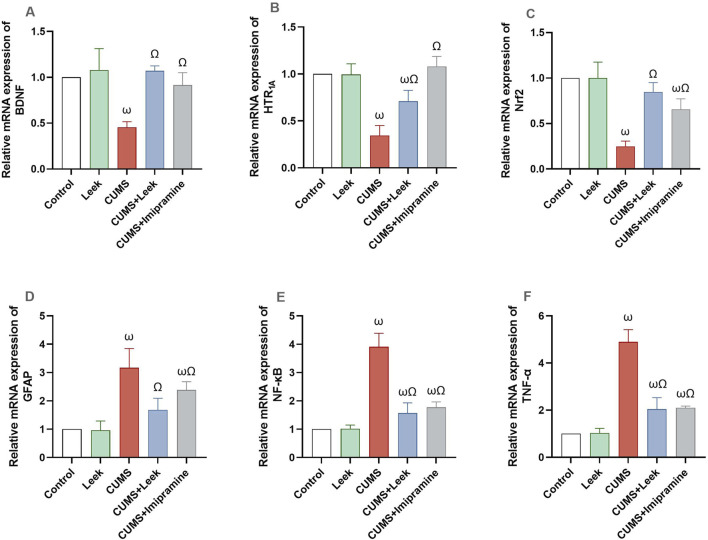
Gene expression analysis of several key genes involved in neuroplasticity, neurotransmission, and neuroinflammation in the cerebral cortex of rats exposed to CUMS and the effects of leek treatment. **(A)** Relative mRNA expression of BDNF, **(B)** Relative mRNA expression of 5-HT_1A_, **(C)** Relative mRNA expression of Nrf2, **(D)** Relative mRNA expression of GFAP, **(E)** Relative mRNA expression of NF-κB, and **(F)** Relative mRNA expression of TNF-α following CUMS exposure and leek treatment. Data are expressed as mean ± SEM (n = 6 rats/group). For statistical significance, ωP < 0.05, compared to Control group; ΩP < 0.05, compared to CUMS group (one-way ANOVA followed by Tukey’s test).

Gene expression analysis revealed significant alterations in key genes associated with neuroplasticity, neurotransmission, and neuroinflammation in the cerebral cortex of rats exposed to CUMS. The CUMS group exhibited marked downregulation of BDNF, 5-HT_1A_, and Nrf2 expression (all ωP < 0.0001), alongside significant upregulation of GFAP (ωP < 0.0001). Pro-inflammatory factors (NF-κB and TNF-α) were also upregulated (both ωP < 0.0001), in comparison to the Control group. Treatment with leek normalized these alterations, significantly upregulating BDNF, 5-HT_1A_, and Nrf2 (all ΩP < 0.0001), while reducing inflammatory factors and GFAP expression (all ΩP < 0.0001), in comparison to the CUMS group. The CUMS + Imipramine group showed comparable therapeutic effects, with significant normalization of all measured genes (ΩP < 0.0001), except for GFAP, which showed a significant but minimal reduction (ΩP < 0.05) (Figure 13).

## 4 Discussion

During the study, we successfully induced depression in male rats using the CUMS model, a well-validated animal model that effectively mimics core depressive symptoms in human, particularly anhedonia (loss of pleasure) (Zheng et al., 2020). Chronic stress is a key contributor to depression, and the CUMS model replicates the persistent exposure to unpredictable, low-grade stressors resembling those encountered in human daily life, making it a valuable tool for depression research (Tang et al., 2025). Behavioral assessments, including the SPT, OFT, and SIT, confirmed the induction of depression-like behaviors, validating the model’s successful establishment. Notably, the treatment with the Egyptian leek (*Allium ampeloprasum* var. *kurrat*) extract demonstrated significant antidepressant-like effects in CUMS-exposed rats, effectively ameliorating depressive-like behaviors. These therapeutic effects were evidenced by behavioral improvements including increased sucrose intake (indicating reduced anhedonia), enhanced exploratory activity, and restored social interaction, all representing core symptom relief in depression. The effects were comparable to those elicited by imipramine, suggesting that the extract may share some mechanistic pathways with standard antidepressant treatments. Additionally, the reduction in anxiety-related behaviors, such as freezing and avoidance, further underscores the potential anxiolytic properties of the leek extract, which could be beneficial for comorbid anxiety and depression. The behavioral improvements were accompanied by significant neurochemical and molecular changes. The multi-target approach of the leek extract addressed the multifactorial pathophysiology of depression, including oxidative stress, neuroinflammation, impaired neuroplasticity and apoptosis.

The brain’s high lipid content, oxygen consumption capacity, and energy demands make it particularly vulnerable to oxidative stress, a key contributor to depression (Barbosa et al., 2020; Bhatt et al., 2020). Prolonged stress exposure stimulates excessive reactive oxygen species (ROS) generation, which disrupts the normal functioning of the antioxidant defense system (Liu et al., 2025). The imbalance between ROS overproduction and antioxidant defenses leads to oxidative stress, further alters neuronal structure and function (Jindal et al., 2015; Liu et al., 2025). The antioxidant enzymes including GSH, GPx, SOD, and CAT combat and protect against oxidative damage by blocking and removing formed free radicals preventing the occurrence of ROS (Barbosa et al., 2020). The high total phenolic and flavonoid content of Egyptian leek extract underscores its potent antioxidant capacity, which aligns with its observed neuroprotective effects. The leek extract enhanced antioxidant defense mechanisms by increasing the activity of key antioxidant enzymes, while significantly reducing levels of NO and MDA, a product of lipid peroxidation. The leek extract was also found to upregulate Nrf2, a critical transcription factor that promotes the expression of these antioxidant enzymes (Ngo and Duennwald, 2022; Subba et al., 2022). This suggests that Egyptian leek activates the Nrf2 pathway, a key regulator of cellular antioxidant defense mechanisms. Nrf2, upon activation, translocates into the nucleus, where it binds to the antioxidant response element (ARE) in the promoter regions of target genes. This binding induces the expression of antioxidant genes, maintaining cellular redox balance, thereby protecting neurons from oxidative stress (Subba et al., 2022). Nrf2 signaling plays a crucial role in antioxidative stress, anti-inflammatory responses and anti-apoptosis, all of which are disrupted in depression (Ngo and Duennwald, 2022; Subba et al., 2022). Via this mechanism, Egyptian leek restored antioxidant defenses, protecting against oxidative damage and contributing to its antidepressant effects.

The leek extract also reduced inflammatory response, supporting its anti-inflammatory properties. This effect is critical, as neuroinflammation contributes to synaptic dysfunction, neuronal atrophy and glial loss, neurotransmitters imbalance, and depressive behaviors (Maeng and Hong, 2019; Hassamal, 2023). The observed downregulation of NF-κB, a central regulator of neuroinflammation, and its downstream pro-inflammatory cytokines suggests inhibition of the NF-κB pathway as a key mechanism. CUMS-induced chronic stress can activate the NF-κB pathway, triggering the expression and release of pro-inflammatory cytokines such as TNF-α, IL-1β, and IL-6. These cytokines stimulate the HPA axis by interacting with glucocorticoid receptors (GR), leading to excessive release of glucocorticoids (GCs). Although GCs typically exert anti-inflammatory effects under normal conditions, their chronic overproduction disrupts feedback mechanisms, thereby sustaining neuroinflammation and contributing to depressive symptoms (Sălcudean et al., 2025). The resulting excess of GCs further activates both microglia and astrocytes, prompting the release of additional pro-inflammatory cytokines that stimulate the HPA axis and sustain neuroinflammation. This creates a vicious cycle of neuroinflammation and HPA axis dysregulation. Excessive microglial activation not only mediates neuroinflammatory responses but also disrupts neurotransmitter homeostasis, altering the balance between excitatory and inhibitory neurotransmitters (Hassamal, 2023; Sălcudean et al., 2025). Meanwhile, astrocyte hyperactivity leads to overexpression of GFAP, a key astrocyte marker (Hassamal, 2023; Sălcudean et al., 2025). NF-κB acts as a transcription factor for GFAP by binding to its promoter and enhancing its expression (Li et al., 2020). While GFAP helps maintain astrocyte structural integrity under normal conditions, its pathological overexpression contributes to depression-associated synaptic dysfunction, mainly through impaired neurotransmitter clearance (Sălcudean et al., 2025). The significant downregulation of GFAP expression observed in the leek-treated group suggests that Egyptian leek may help restore the neural microenvironment by attenuating glia-mediated neuroinflammation. This conclusion was further supported by immunohistochemistry analysis, which revealed a marked decrease in GFAP-positive cells in the cortical tissue of CUMS-exposed rats following leek treatment. These findings indicate that leek exerts a glioprotective effect, effectively counteracting the CUMS-induced upregulation of GFAP and mitigating associated glial activation. By targeting the NF-κB pathway, leek’s anti-inflammatory and neuroprotective effects offer a promising strategy for mitigating the neurobiological mechanisms of depression. Inhibiting NF-κB can enhance GR transcription, reduce elevated GC levels, and modulate HPA axis hyperactivity, thereby breaking the cycle of stress and inflammation (Liu et al., 2023).

At the neurochemical level, the leek extract restored the levels and balance of monoamine neurotransmitters (5-HT, DA, NE), which are critical for synaptic transmission and neural communication and are often disrupted in depression (Cui et al., 2024). Sustained microglial and astrocytic activation causes a continuous neuroinflammatory response, associated with increased activity of the HPA axis. This creates a neurotoxic environment, further exacerbating glial activation and disrupting neurotransmitter homeostasis. The resulting imbalance between excitatory and inhibitory neurotransmission impairs synaptic plasticity, ultimately contributing to mood dysregulation (Yan et al., 2022; Sălcudean et al., 2025). Prolonged hyperactivity of the HPA axis disrupts all three major monoamine systems in depression. First, it reduces 5-HT levels in mood-regulating areas such as the prefrontal cortex and hippocampus. Dysfunctional serotonergic signaling, including downregulation of 5-HT_1A_, further contributes to depressive symptoms. Second, chronic GCs exposure impairs dopaminergic function by reducing both DA synthesis and D2 receptor signaling, thereby disrupting reward processing and promoting anhedonia (Sălcudean et al., 2025). Third, the noradrenergic system, which is critical for arousal and stress responses (Gu et al., 2023), shifts from adaptive acute activation (promoting alertness) (Sălcudean et al., 2025) to maladaptive chronic states characterized by anxiety, reduced NE synthesis and release (Gu et al., 2023), and a strong correlation with depression severity, completes the triad of monoamine dysfunction that sustains depressive pathophysiology (Sălcudean et al., 2025). By restoring monoamine concentrations and enhancing their synaptic function, leek extract may exert an antidepressant effect. Additionally, leek modulated excitatory-inhibitory balance by reducing excitatory amino acids (Glu, Asp) while enhancing inhibitory signals (GABA, Gly)—along with its distinct impact on ATP levels, suggesting a protective role against neurotoxicity, a hallmark of depressive pathology (Hassamal, 2023). As the dominant excitatory neurotransmitters, Glu and Asp critically regulate synaptic signaling and plasticity. When dysregulated, their excessive extracellular accumulation triggers excitotoxic cascades, a well-established mechanism in depression, and ultimately cell damage (Wang L. et al., 2024). Conversely, GABA and Gly are the primary inhibitory amino acid neurotransmitters and play key roles in preventing excitotoxicity, promoting neuroprotection, and supporting neurotrophic processes (Wang L. et al., 2024). This function becomes particularly important in depression, where disruptions in GABAergic and glycinergic signaling have been observed (de Bartolomeis et al., 2020; Sălcudean et al., 2025). Restoration of ATP levels by the leek extract further supports its antidepressant potential. ATP is critical for maintaining ion gradients necessary for neuronal function, supporting neurotransmitter vesicular transport and release and supporting overall neuronal activity. Its deficiency, particularly in astrocytes, has been linked to depressive pathology, while sufficient ATP availability may help preserve neuronal function and alleviate symptoms (Wang K. et al., 2024).

At the molecular level, leek extract demonstrated a pronounced impact on neuroplasticity by upregulating BDNF. This conclusion was supported by both increased BDNF gene expression and immunohistochemical evidence showing restoration of BDNF-positive cells in cortical tissue of CUMS-exposed rats, demonstrating leek’s neuroprotective effect against stress-induced BDNF downregulation. These findings collectively suggest that Egyptian leek activates the BDNF/TrkB/CREB pathway, a critical signaling cascade for neuroplasticity and neuronal survival (Gong et al., 2024). Chronic stress suppresses this pathway, leading to impaired neuronal growth, differentiation, and survival, resulting in impaired neuroplasticity and eventually triggering depressive symptoms (A. et al., 2025). Numerous studies have shown that BDNF alleviates depression symptoms through high-affinity binding to tropomyosin receptor kinase B (TrkB). This binding triggers TrkB autophosphorylation and initiating downstream signaling pathways, including the Phosphatidylinositol 3-kinase (PI3K)/protein kinase B (PKB also known as AKT) or PI3K/Akt pathway (Yang et al., 2020). Upon activation, PI3K phosphorylates phosphatidylinositol-4,5-bisphosphate (PIP2) to phosphatidylinositol-3,4,5-trisphosphate (PIP3). PIP3 then recruits AKT, promoting its phosphorylation and activation (Guo et al., 2024; Kumar et al., 2025). Once activated, AKT can promote the phosphorylation and activation of cAMP-response element binding protein (CREB), a transcription factor (Guo et al., 2024). Activated CREB binds to the cAMP response element (CRE) in target gene promoters, including the BDNF gene itself (A. et al., 2025), thereby enhancing synaptic connections, supporting neuronal differentiation, proliferation, maturation, and nutrition, and promoting the synthesis and release of neurotransmitters (Tang et al., 2022). BDNF plays a vital role in maintaining the development and function of serotonergic, dopaminergic, glutamatergic, GABAergic, and cholinergic neurons, thereby ensuring efficient synaptic transmission and neural communication (Colucci-D’amato et al., 2020; Shkundin and Halaris, 2023). Disruption of the BDNF/TrkB/CREB pathway and subsequent BDNF downregulation leads to broader neurotransmission dysregulation (Koh et al., 2017; Lin et al., 2018; Colucci-D’amato et al., 2020). Conversely, reduced levels of monoamine neurotransmitters and imbalances in excitatory and inhibitory amino acids can further decrease BDNF levels (Han et al., 2024; Wang L. et al., 2024), creating a bidirectional relationship between neurotrophic support and neurotransmitter homeostasis. Additionally, elevated AChE activity reduces acetylcholine and BDNF levels, exacerbating synaptic and cholinergic dysfunction. Therefore, activation of the BDNF/TrkB/CREB pathway and suppression of AChE activity may represent complementary strategies for improving neurotransmission and alleviating depressive symptoms (Koh et al., 2017; Colucci-D’amato et al., 2020).

The extract’s neuroprotective effects extend beyond BDNF-mediated neuroplasticity to include robust anti-apoptotic actions, counteracting CUMS-induced neuronal damage and promoting cell survival, likely through the PI3K/AKT pathway, as evidenced by modulation of apoptosis-related proteins; increased levels of BCL-2 and decreased levels of BAX and caspase-3. Disruption of the PI3K/AKT signaling pathway in mood-regulating brain areas has been linked to depression, as this pathway is essential for suppressing neuronal apoptosis—a key pathological feature of the disorder. Conversely, enhanced activation of PI3K/AKT signaling has shown rapid and sustained antidepressant effects (Guo et al., 2024). The restoration of normal neuronal morphology observed in histopathological analysis further supports this mechanism. The balance between pro- and anti-apoptotic proteins is crucial for maintaining neuronal integrity (Mustafa et al., 2024), which is disrupted by chronic stress. Reduced Bcl-2 levels and elevated Bax expression disrupt mitochondrial membrane integrity, initiating the caspase cascade. This process leads to the activation of Caspase-3, a key driver of mitochondria-mediated apoptosis (Hong et al., 2024; Wang et al., 2025a). Activation of the PI3K/AKT pathway triggers a cascade of downstream signaling events that ameliorate cellular damage, contributing to neuroprotection. Once activated, PI3K stimulates and activates Akt, which helps inhibit apoptosis by preserving the caspase cascade and enhancing the expression of anti-apoptotic proteins (Guo et al., 2024; Wang et al., 2025a). These findings underscore the therapeutic potential of restoring PI3K/AKT activity to enhance neuroprotection and alleviate depressive symptoms.

Molecular docking analysis identified flavonoids as the primary bioactive compounds mediating the effects of leek extract. While imipramine showed moderate binding affinities in docking studies, the flavonoids in leek extract exhibited stronger and more complex interactions with target proteins. Although sulfur compounds dominated the extract’s chemical profile, they appeared to play a minor role in its antidepressant effects. The observed pharmacodynamic effects were principally attributed to flavonoid and polyphenol components, which seemed to function as receptor agonists—contrasting with imipramine’s antagonistic mechanism. These findings position Egyptian leek extract, particularly its flavonoid constituents (e.g., quercetin-7-O-rutinoside, luteolin 7-O-glucoside), as a promising multi-target therapeutic candidate for depression.

The outcomes of this study on Egyptian leek align closely with previous studies on other Allium species, such as garlic and onions, which have also demonstrated potential antidepressant effects in rodent models (Rahmani et al., 2020; Chen et al., 2022; Alharbi et al., 2025). This consistency across studies highlights the potential of Allium species as a source of natural antidepressants and supports further investigation into their therapeutic potential for depression.

## 5 Conclusion

Our study demonstrates that Egyptian leek extract alleviates depression-like symptoms in CUMS-exposed rats through a multi-target mechanism involving antioxidant, anti-inflammatory, anti-apoptotic, and neurotrophic effects. According to molecular docking analysis, flavonoid constituents, particularly quercetin-7-O-rutinoside and luteolin-7-O-glucoside, may play a key role in mediating these effects. However, molecular docking provides only predictive insights and does not confirm biological activity. Therefore, further studies are needed to validate the molecular docking findings and confirm the roles of these compounds. These findings underscore the therapeutic potential of Egyptian leek and contribute to the growing body of evidence supporting the use of natural products in mental health treatment. While this study focused on a single effective dose (100 mg/kg), future work should include dose-response studies to identify optimal therapeutic ranges and evaluate safety thresholds. Detailed pharmacokinetic studies are needed to determine the bioavailability and metabolism of its bioactive compounds, which are critical for evaluating its clinical potential. Future investigations should also explore the role of epigenetic regulation and gut-brain axis modulation in its antidepressant effects. Moreover, investigating the synergistic potential of Egyptian leek in combination with existing antidepressants could provide valuable insights for integrated therapeutic strategies.

## Data Availability

The original contributions presented in the study are included in the article/Supplementary Material, further inquiries can be directed to the corresponding authors.
