# Supplementary material for: Multi-target approach of Egyptian leek extract in ameliorating depressive-like behavior in rats exposed to chronic unpredictable mild stress
Source: Front Pharmacol. 2025 Aug 4;16:1621762. doi: 10.3389/fphar.2025.1621762 (PMC12358850; doi:10.3389/fphar.2025.1621762)
Supplement: Supplementary file 1 [file DataSheet1.pdf]

Multi-Target Approach of Egyptian Leek Extract in Ameliorating Depressive-Like Behavior in Rats Exposed to Chronic Unpredictable Mild Stress

Mai M.S. Mahmoud[1], Amina E. Essawy[2], Ahmed A. Soffar[2], Ahmed H.I. Faraag[3], Mohamed A. Dkhil[4], Omar A. Ahmed-Farid[5], Manal F. El-khadragy[6], Ahmed E. Abdel Moniem[4,7,*]

1. Euro-Mediterranean Program in Neuroscience and Biotechnology, Faculty of Science, Alexandria University, Alexandria, Egypt
2. Zoology Department, Faculty of Science, Alexandria University, Alexandria, Egypt
3. Botany and Microbiology Department, Faculty of Science, Helwan University, Cairo, Egypt
4. Zoology and Entomology Department, Faculty of Science, Helwan University, Cairo, Egypt
5. Physiology Department, Egyptian Drug Authority, Giza, Egypt
6. Department of Biology, College of Science, Princess Nourah Bint Abdulrahman University, Riyadh, Saudi Arabia
7. Al-Ayen Scientific Research Center, Al-Ayen Iraqi University, AUIQ, An Nasiriyah, P.O. Box: 64004, ThiQar, Iraq

* **Correspondence:** aest1977@hotmail.com; ahmed_abdelmoneim@science.helwan.edu.eg

Supplementary data: The chromatogram and standard plots for the HPLC detection method for section 3.6. Changes in Free Amino Acids, Monoamines, and Purinergic Metabolites

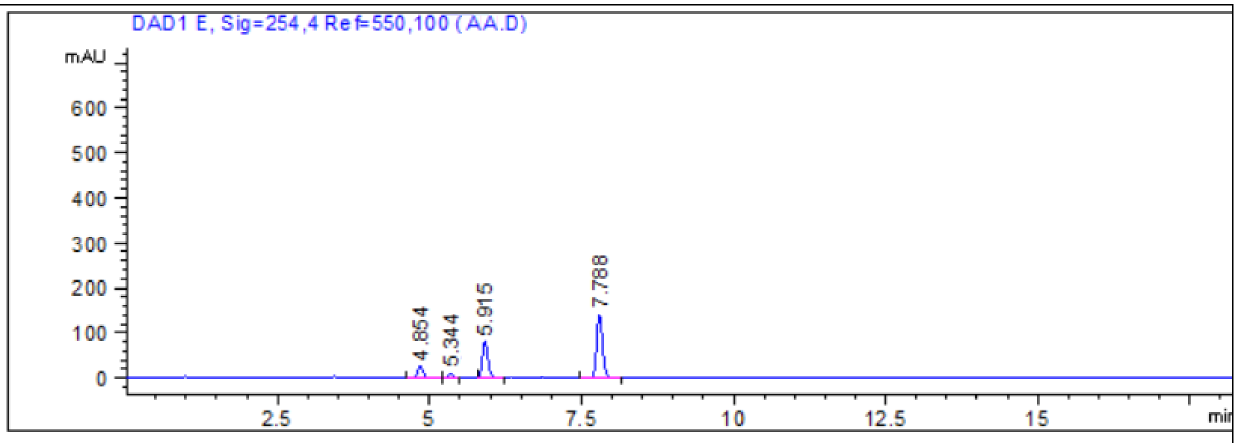

Figure S1: HPLC chromatogram of the amino acid standard mixture (Asp, Glu, Gly, and GABA), showing retention times of 4.8, 5.3, 5.9, and 7.7 minutes, respectively.

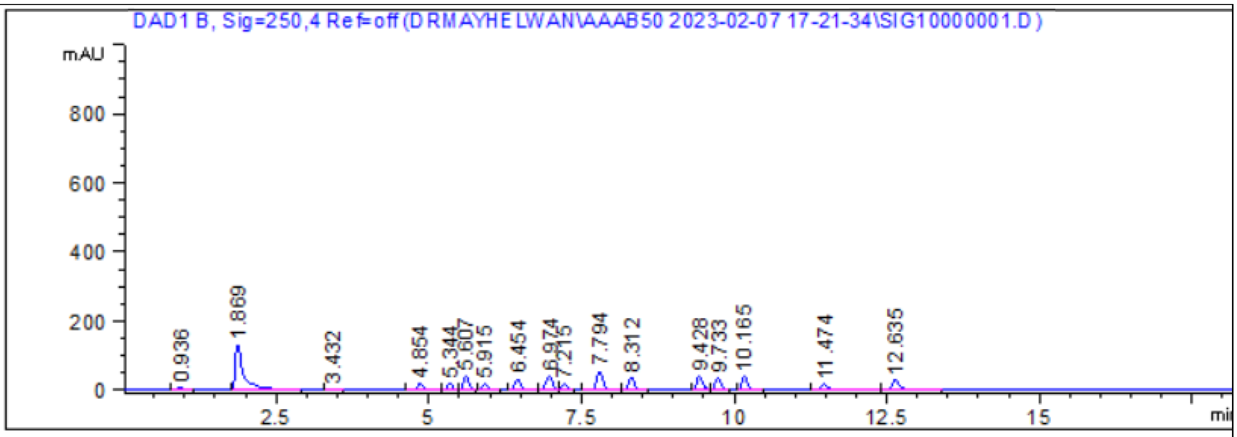

Figure S2: Representative HPLC chromatogram of the brain sample, confirming the detection of Asp, Glu, Gly, and GABA based on their respective retention times (4.8, 5.3, 5.9, and 7.7 minutes).

A

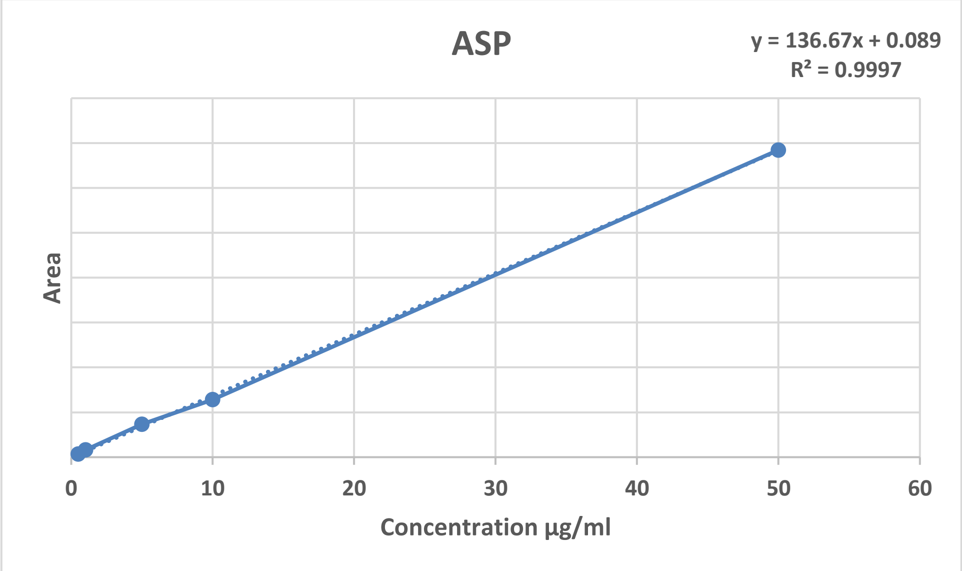

B

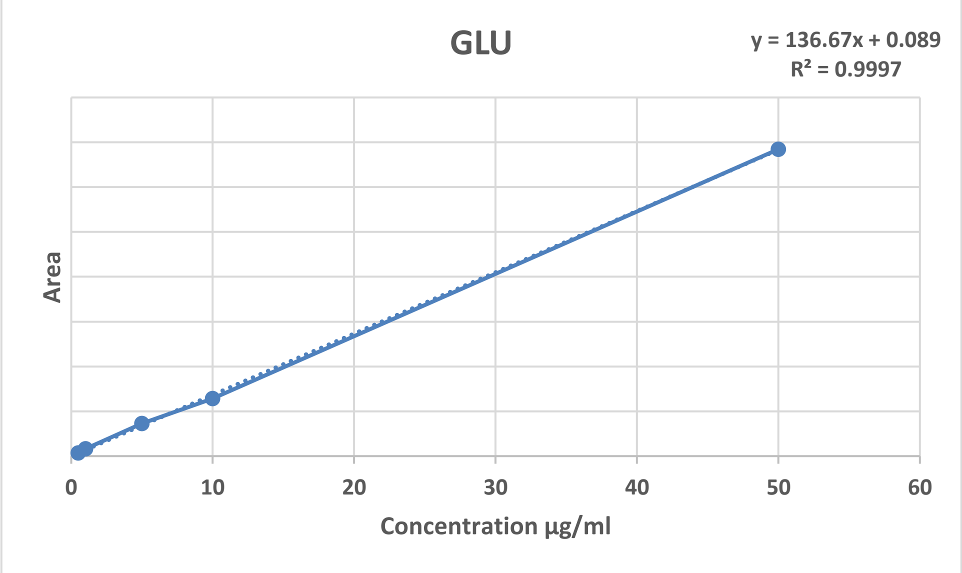

C

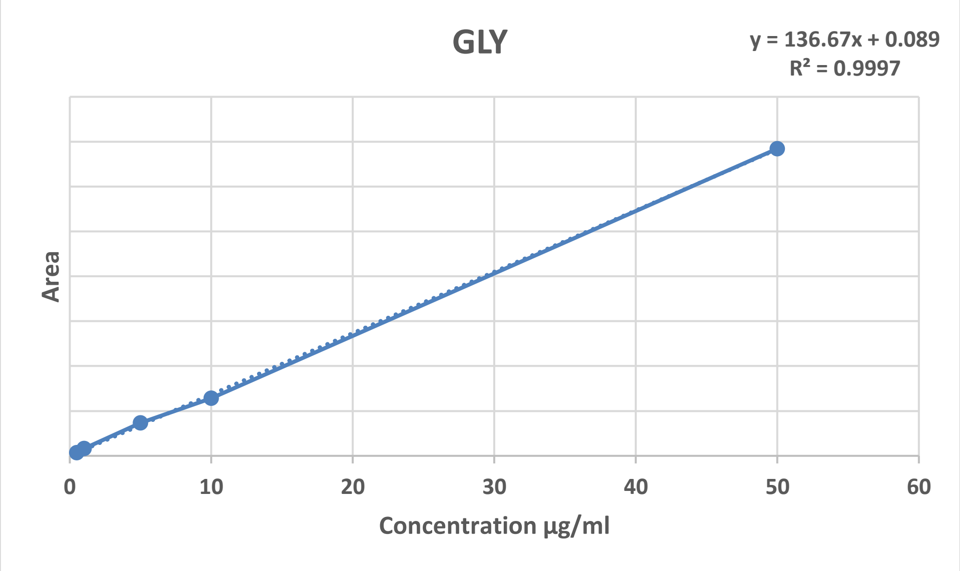

D

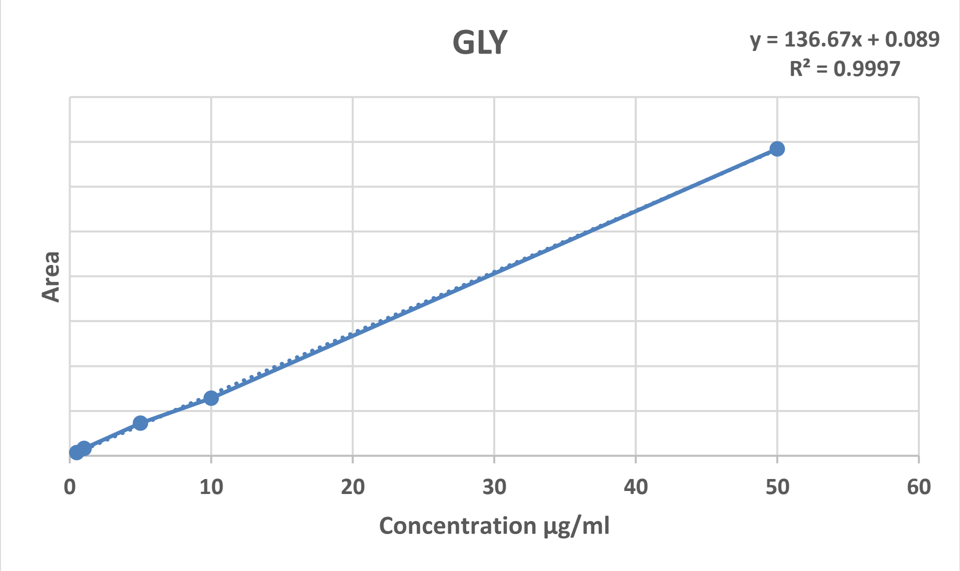

Figure S3: Standard curves for each amino acid in the mixture (ASP, GLU, GLY, and GABA), including the corresponding α and β coefficients.

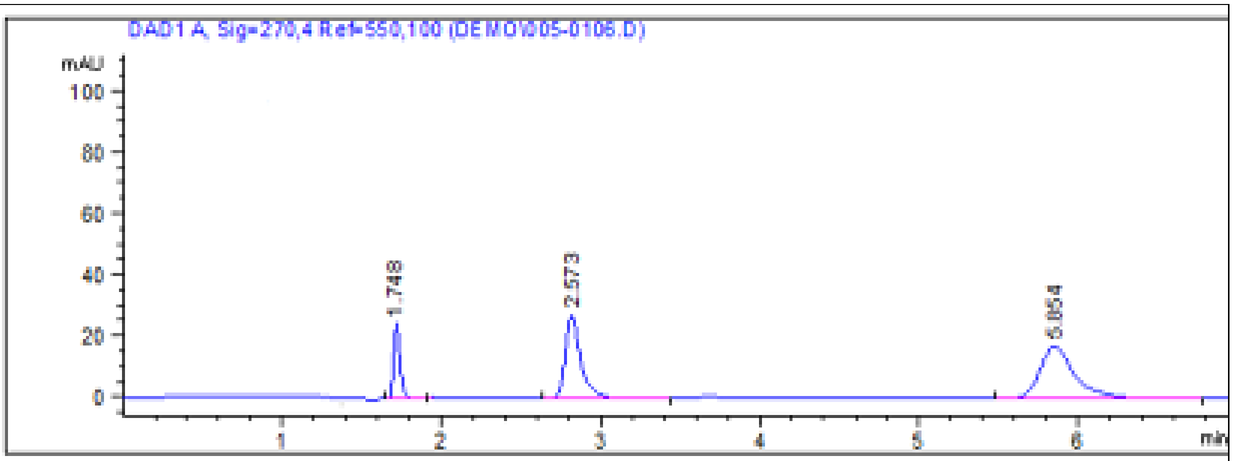

Figure S4: HPLC chromatogram of the monoamines standard mixture (NE, DA, and 5HT), showing retention times of 4.8, 5.3, 5.9, and 7.7 minutes, respectively.

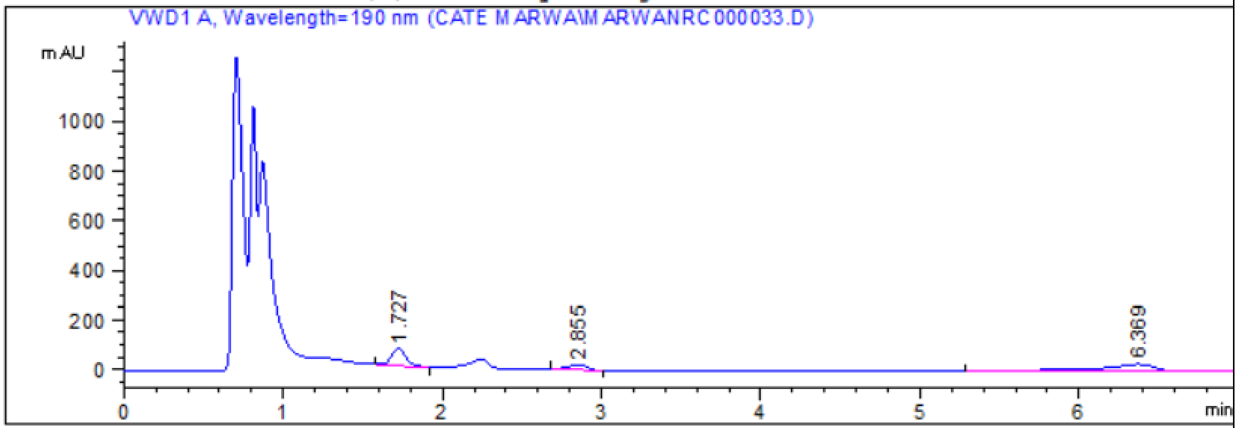

Figure S5: Representative HPLC chromatogram of the brain sample, confirming the detection of NE, DA and 5HT based on their respective retention times (1.7, 2.8, and 6.3 minutes).

A

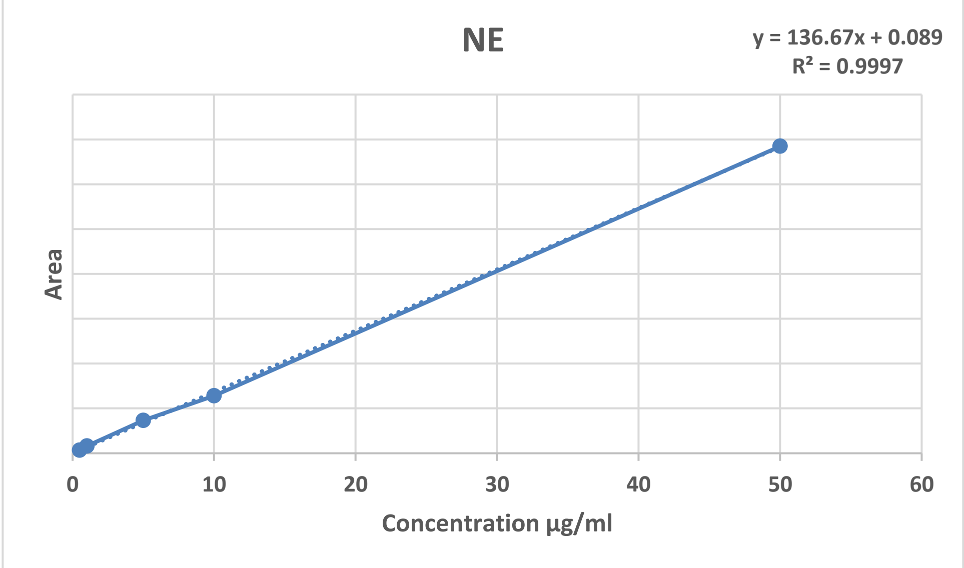

B

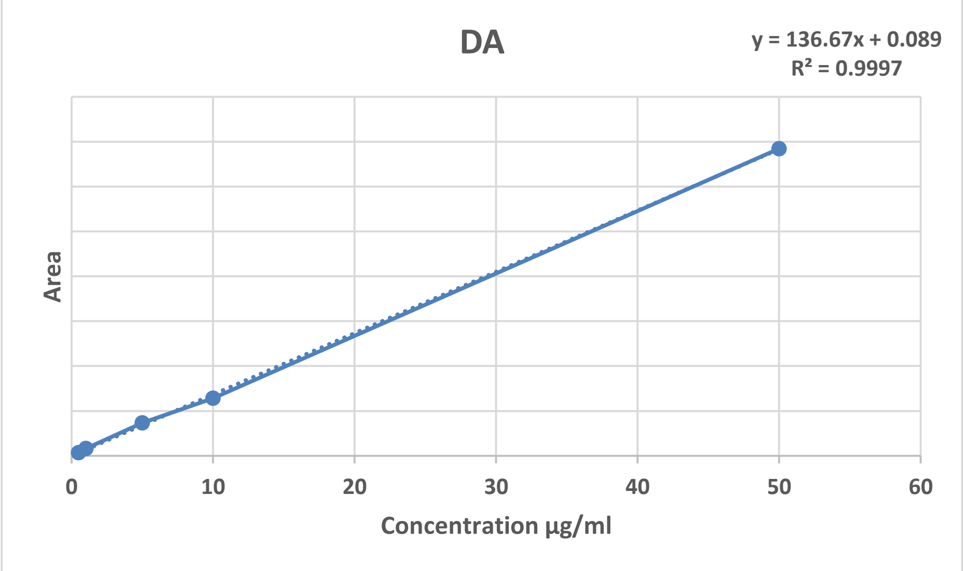

C

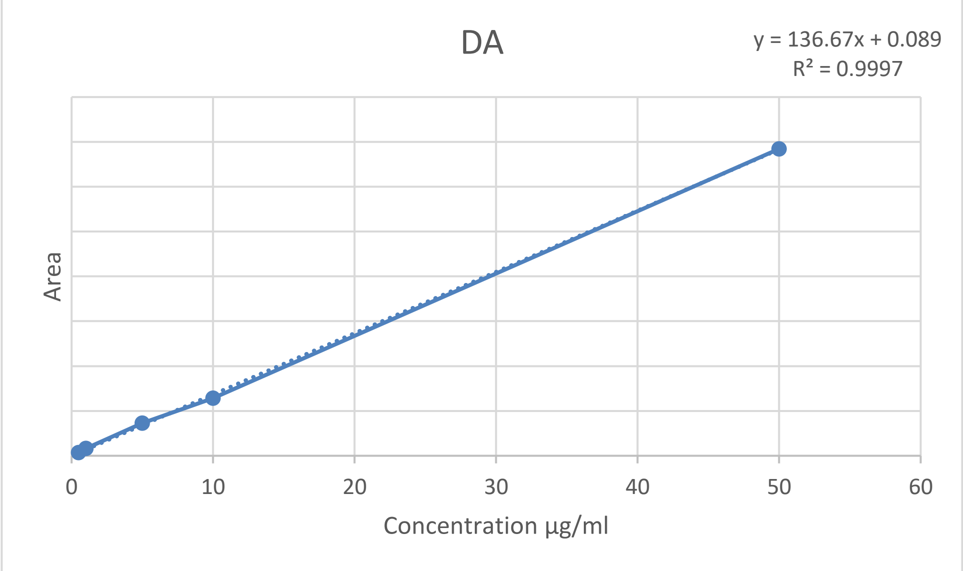

Figure S6: Standard curves for each monoamines in the mixture (NE, DA, and 5HT), including the corresponding α and β coefficients.

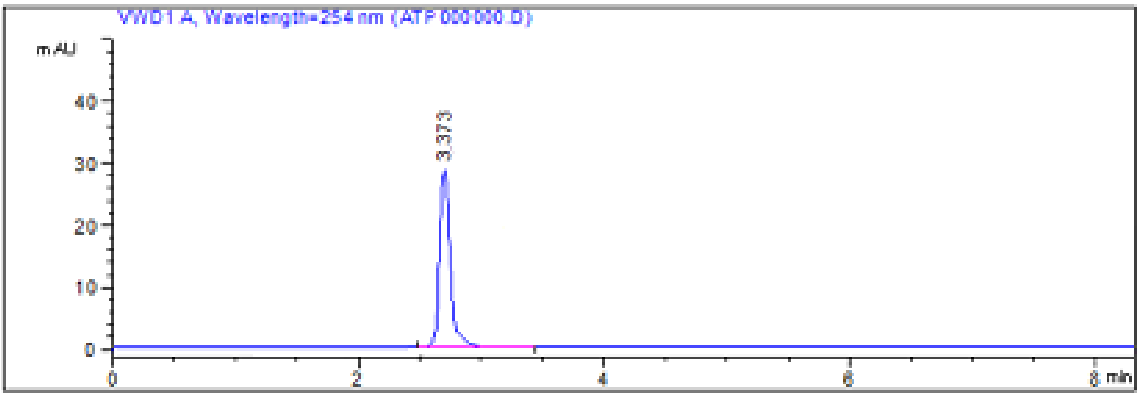

Figure S7: HPLC chromatogram of the ATP standard, showing at retention times of 3.3 minutes.

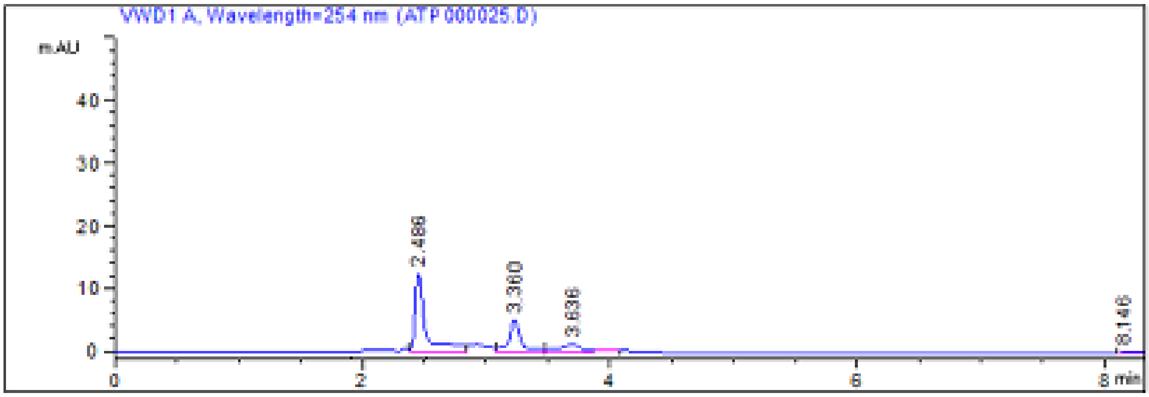

Figure S8: HPLC chromatogram of the ATP barin sample, showing at selected retention times of 3.3 minutes.

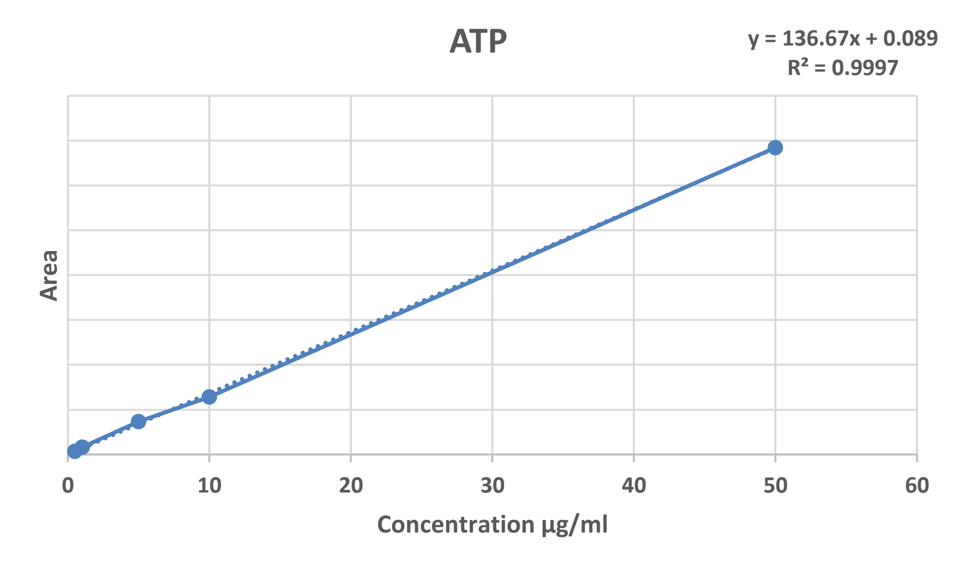

Figure S9: Standard curves for purinergic compound (ATP), including the corresponding α and β coefficients.
